# Introducing assisted reproductive technologies in The Gambia, a survey on the perspectives of Gambian healthcare professionals and medical students

**DOI:** 10.1186/s12913-023-09171-7

**Published:** 2023-02-28

**Authors:** Haddy Bittaye, Jason P. Mooney, Anna Afferri, Julie Balen, Vanessa Kay

**Affiliations:** 1 Wellingara, Serekunda, The Gambia; 2 Institute of Immunology and Infection Research, School of Biological Sciences, University of Edinburgh, Edinburgh, UK; 3 School of Health and Related Research, University of Sheffield, Sheffield, UK; 4 School of Medicine, University of Dundee, Dundee, UK

**Keywords:** Infertility, ART, LMIC, IVF, The Gambia, Survey, Healthcare professionals, Medical students

## Abstract

**Background:**

Infertility remains a global reproductive health burden with the highest prevalence in low and middle-income countries. In sub-Saharan Africa, the ability to procreate holds great societal importance. Couples, and particularly women, with infertility can face devastating challenges, leading to social stigma, isolation and/or divorce. However, attention to addressing infertility is lacking in sub-Saharan Africa. In The Gambia, where this study is based, little is known about the potential for introduction of assisted reproductive technologies (ART) in the public health sector.

**Methods:**

A quantitative survey was conducted using detailed questionnaires on infertility services available, staff knowledge, perceived barriers, and personal motivation to support assisted reproductive technologies. Data was collected electronically between April and June 2021 from healthcare providers (n = 70) in eleven health facilities throughout the country, as well as from medical students (n = 55) enrolled at The University of The Gambia.

**Results:**

Basic infertility services were found to be lacking in the rural areas. Furthermore, 39% of staff (n = 27) providing fertility care had not receive any formal training on the topic. However, 91% of staff (n = 64) showed interest in acquiring additional knowledge and had a positive attitude towards supporting the introduction of ART. Perceived challenges of doing so included: (i) the competing importance of other health priorities; and (ii) religious and cultural barriers.

**Conclusion:**

This survey highlights that expansion of infertility services is needed, especially in rural areas. Staff perceived the introduction of ART as important, but this should be coupled with specialized training, as most medical staff had not received any formal infertility training. Future care providers (current medical students) showed both interest in ART and reported having received some basic training in infertility management. Given the reported lack of infrastructure and services, additional targeted investment in infertility care, including ART, will be needed to improve reproductive health for all, countrywide.

**Supplementary Information:**

The online version contains supplementary material available at 10.1186/s12913-023-09171-7.

## Background

In Sub-Saharan Africa, the prevalence of involuntary childlessness (infertility) is high. However, services to help address this problem are inconsistent or lacking – including in the West African country of The Gambia, where this study was conducted. There is currently limited information on the infrastructure available and the level of knowledge and training among healthcare providers in the country to help address this issue. To address this gap in knowledge, we conducted a survey with health staff from different hospitals and with medical students at the University of The Gambia. The survey focused on the reported availability of services as well as participants’ knowledge and training in both basic and advanced treatments for infertility. We found a major lack of infertility care services in rural areas. Furthermore, staff knowledge about infertility was minimal, and there was little knowledge about more advanced infertility care services, such as in vitro fertilization (IVF). The survey also found that staff and students perceived other health priorities and religion as major barriers for ART introduction. Staff noted that government funding for infertility treatment is minimal. In conclusion, this survey found a lack of reported infrastructure for infertility in rural areas of The Gambia, as well as a lack of formal training by staff. However, both staff and students showed interest in the potential introduction of ART. Overall, this survey highlights that more investment and training is needed to implement changes in order to modernize reproductive healthcare in The Gambia, in particular for those who face infertility.

**Supplementary Information:**

The online version contains supplementary material available at 10.1186/s12913-023-09171-7.

## Background

Infertility is a major reproductive health problem that impacts approximately 10–25% of reproductive age couples globally [1]. The highest prevalence of infertility is seen in low and middle-income countries (LMIC), including across sub-Saharan Africa (SSA) [2]. For example, infertility is estimated at 11.8% in rural Ghana [3] and 27.6% among three hospitals in Ethiopia [4]. In The Gambia, infertility appears to be on the rise, with an estimated prevalence of 9% in 1998 [5] and 12% in 2012 [6]. In SSA, where health systems are still fragile, claims of overpopulation, competing importance of other health priorities and limited health budgets may inhibit the prioritization of infertility services [7, 8]. Yet, this combination of high rates of infertility, competing health needs and a lack of appropriate provision of services has a major impact on individuals living with infertility across SSA.

Infertility can cause devastating consequences to couples, especially women, including severe physical and psychological distress [9–12]. For women, this can lead to depression, reduced sexual interest, self-blame, and a loss of social status – resulting in significant social isolation and stigma [13]. Women with infertility are also at an increased risk of HIV infection compared to fertile women (18.2% vs. 6.6% respectively) [14], because of an increase in sexual partners in the pursuit of a child. For married women, unsuccessful attempts at conception can lead to marital instability, increased extramarital affairs [15] and divorce [10]. Therefore, improving fertility care in SSA is imperative as it can impact society beyond the conception of a child and can have lasting impacts on gender equality and the reproductive rights of individuals across societies.

One method to address infertility is medically assisted reproduction interventions specific to ART which includes, but not limited to, in vitro fertilization (IVF) and intracytoplasmic sperm injection (ICSI). In 2014, The International Committee Monitoring Assisted Reproductive Technologies (ICMART) reported a total of 1,648,000 ART cycles performed in 76 participating countries globally, with Africa accounting for just 1% of the total cycles performed [16]. In The Gambia, a recent study illustrated that only 66% of health facilities offer any form of infertility treatments such as ovulation induction, tubal ligation reversal, and intrauterine insemination (IUI) [17]. However, no ART services are currently available. Given that male factor infertility and tubal damage by chronic and untreated sexually transmitted diseases (STI) are the leading causes of infertility in The Gambia [18], which can be treated by ART, its introduction could assist Gambians achieve a pregnancy and live birth. This study serves as a baseline to explore the understanding, interest, and willingness of current and future health care providers to support the introduction of ART in the country. In addition, it will serve as a guide to stakeholders (e.g. Gambian Ministry of Health and policy makers) on these perceptions of Gambian healthcare professionals prior to any future introduction of ART in The Gambia.

## Methods

### Study design

This study was designed in Spring 2021 to operate in a remote manner due to the COVID-19 pandemic. Therefore, an online quantitative survey approach was found to be most suitable design for this study. Questionnaires were designed using Google Forms for staff (43 questions) and for medical students (22 questions) by the lead author (HB) in Dundee, as part of her MSc dissertation. The questionnaires were divided in nine sections, specifically: (i) introductory note; (ii) participants’ demographic; (iii) current infertility services provided; (iv) available infrastructure; (v) knowledge and motivation to support ART services; (vi) infertility training; (vii) barriers to ART introduction; (viii) funding for infertility care; and (ix) informed consent. Students were not surveyed on sections relating to current infertility services provided and available infrastructure, as they were not practicing medicine at the time of the survey, so their responses to these sections may have been inaccurate. Prior to roll out, the questionnaire was piloted on seven medical students from the University of Dundee and three health care providers from The Gambia, with revisions being made based on these results. No validation of the survey content was conducted. Responses to each question was optional. The survey’s landing page included an introductory note explaining the rationale of the study, guidance on study sections and estimated time of completion. Further, it included a statement of infertility in The Gambia as a current reproductive health problem and ART as an effective treatment for some forms of infertility that is not currently available in the country. Questions were formatted as multiple choice or a five-point Likert scale (e.g. least likely (not important) to most likely (very important)) [19]. Questionnaires and all communications were sent out in English, the official language of The Gambia. The ethical approval declarations, the participant information sheet, and questionnaires are available as PDFs in the supplementary files.

### Study setting and survey respondents

This study was conducted in The Gambia from 15th April 2021 to 20th June 2021. Recruitment of staff participants occurred in two phases; snowball, followed by random sampling. First, survey invitations were sent out via email by the Directors of the three largest hospitals, located in Banjul, Brikama and Kanifing, to share with medical staff in their facilities. Second, participants were also recruited randomly through The Gambian healthcare workforce WhatsApp groups, such as the ‘Association of Resident Doctors The Gambia (GARD)’ and the ‘National Association of Gambia Nurses and Midwives’. Survey invitations did not target any specific medical subspecialty (e.g. gynecologists) or hospitals with known infertility care services. The sampling was purposefully broad and aimed to reach across all demographics of healthcare professionals. Medical students (years 5–7) from The University of The Gambia (UTG) were recruited by a random sampling method through the WhatsApp group ‘Unigamsa’. Specific inclusion criteria included those aged 18 or over and either medical students (years 5–7) or in-service medical staff. In total, 150 staff members and 90 students were sent the survey link. This information was visible within the WhatsApp group channels.

**Fig. 1:**
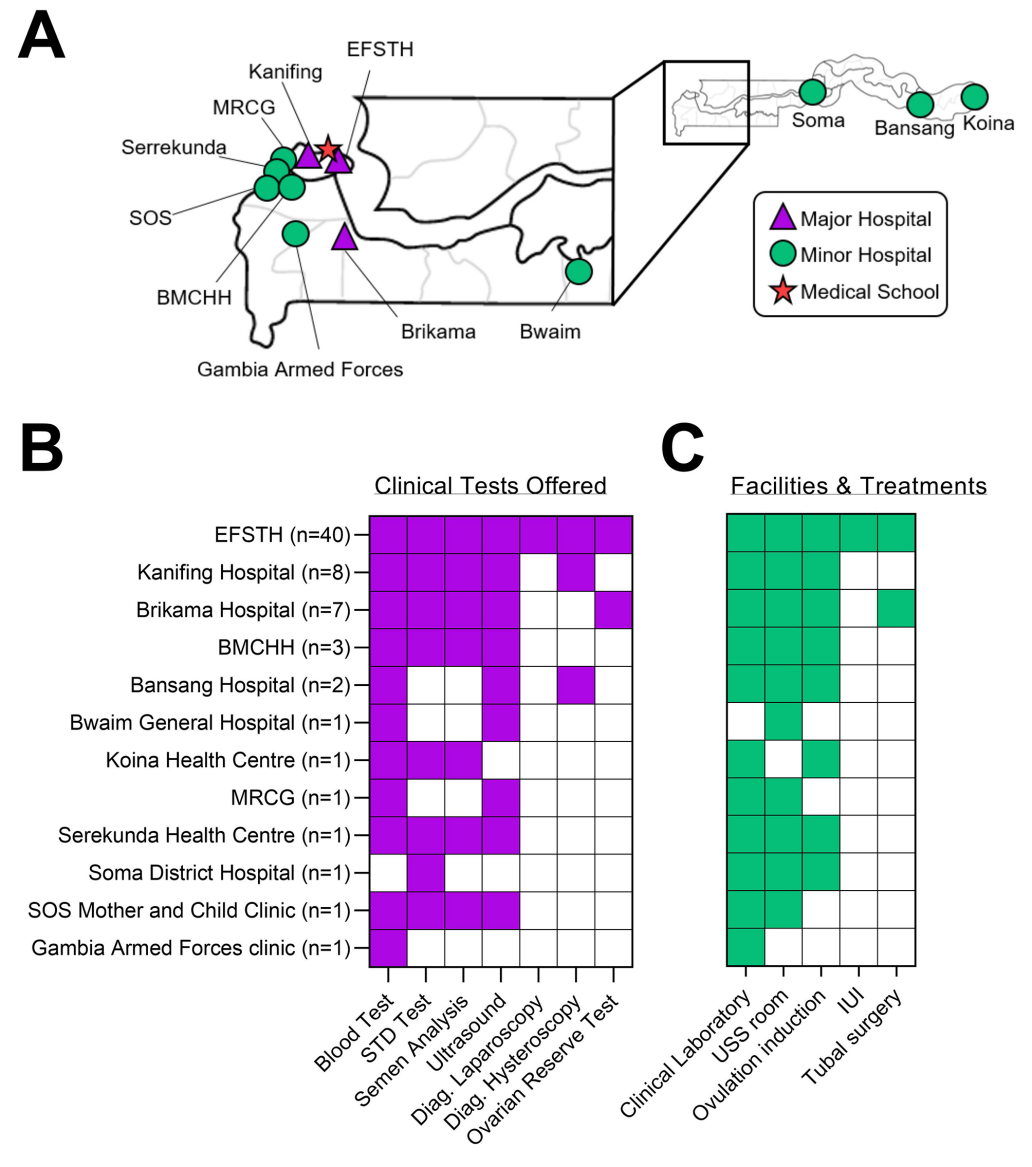
The reported infrastructure and services available for infertility in hospitals in The Gambia, as informed by survey respondents. (**A**) The University of The Gambia Medical School, major survey hospitals (Edward Francis Small Teaching Hospital (EFSTH), Kanifing General Hospital, Brikama Hospital) and minor hospitals (Bundung Maternal Care Health Hospital (BMCHH), Serrekunda Health Centre, Soma District Hospital, Bwaim General Hospital, Bansang Hospital, Medical Research Council Gambia Unit, SOS mother and child Clinic and Gambia Armed Forces Clinic). Hospitals were classified as ‘major’ or ‘minor’ based on their size. (**B**) Clinical investigations offered in each hospital, with the number of survey respondents per location indicated. (C)The facilities and treatments available in each hospital needed for infertility care. (**B-C**) The clear boxes represents unavailability

Invitations were received by participants in 12 health facilities throughout The Gambia; of which three facilities were, for the purpose of this study, classified as ‘major’ referral hospitals in the urban area of Kombo (Kanifing, Brikama, and the Edward Francis Small Teaching Hospital (EFSTH)) and the remaining 9 classified as ‘minor’ hospitals, all distributed across the West and Upper regions of the country (hospitals shown in Fig. 1A). Major hospitals are the largest treatment centres in The Gambia, located in dense urban areas, while minor hospitals are smaller facilities and/or located in rural areas.

### Data analysis

Raw data was transferred to Excel using Google Form for analysis. Graph generation was performed using GraphPad Prism version 8.2.1.

## Results

A total of 240 individuals (150 staff and 90 medical students) received the survey link inviting them to take part in the study, and 125 individuals (70 staff and 55 medical students) responded to the questionnaire (52% response rate). The demographic data are summarized in Table 1, with 124 providing demographic data. The age range was from 18 to 54 years, with the majority being 25–34 years for both groups (n = 94, 76%). Among the respondents, there were more females than males (n = 67 vs. n = 57, respectively). The majority of participants were Muslims (n = 106, 85%). In terms of nationality, 94% of the respondents were Gambian.

**Table 1:** Demographic characteristics of the survey participants

		Staff (n = 70)	Students (n = 55)	
Gender	Male (n, %)	32 (46%)		25 (45%)
	Female (n, %)	38 (54%)		29 (53%)
	Prefer not to say (n, %)	0 (0%)		1 (2%)*
Age	18–24 (n, %)	2 (3%)		13 (24%)
	25–34 (n, %)	55 (79%)		39 (71%)
	35–44 (n, %)	12 (17%)		2 (4%)
	45–54 (n, %)	1 (1%)		0 (0%)
Nationality	Gambian (n, %)	66 (94%)		50 (91%)
	Other (n, %)	4 (6%)		5 (9%)
Religion	Muslim (n, %)	61 (87%)		45 (82%)
	Christian (n, %)	9 (13%)		9 (16%)
	Prefer not to say (n, %)	0 (0%)		1 (2%)

*one student declined to provide some demographic data

### Reported infrastructure and services for infertility in hospitals in the Gambia

To inform on future ART provision, participants were asked about the availability of infertility care services. Of 67 respondents, the majority worked at EFSTH (n = 40, 60%), followed by Kanifing (n = 8, 12%), Brikama (n = 7, 10%), and fewer respondents from the remaining 9 smaller ‘minor’ hospitals. Regarding who provides fertility care, gynecologists are identified as the main fertility care providers in their hospitals (n = 57, 85%). The overall responses indicate EFSTH offered all the clinical investigations for infertility care. Over half of the tests are offered in Kanifing, Brikama, Bundung Maternal and Child Health Hospital (BMCHH), Bansang, Serrekunda and the SOS Mother and Child Clinic (SOS) respectively. Whereas, the remaining offer fewer clinical tests (Fig. 1B). For available facilities, about half of the health centers have all the facilities required for infertility care and the remaining have at least 2 available facilities (Fig. 1C). When asked about infertility treatments, responses showed that EFSTH reportedly has about half of the treatments currently available. By contrast, the remaining hospitals offer only one treatment option or none, with the exception of Brikama Hospital which offers two treatment options available (ovulation induction and tubal surgery) (Fig. 1C).

### Infertility patients in the Gambia

**Fig. 2:**
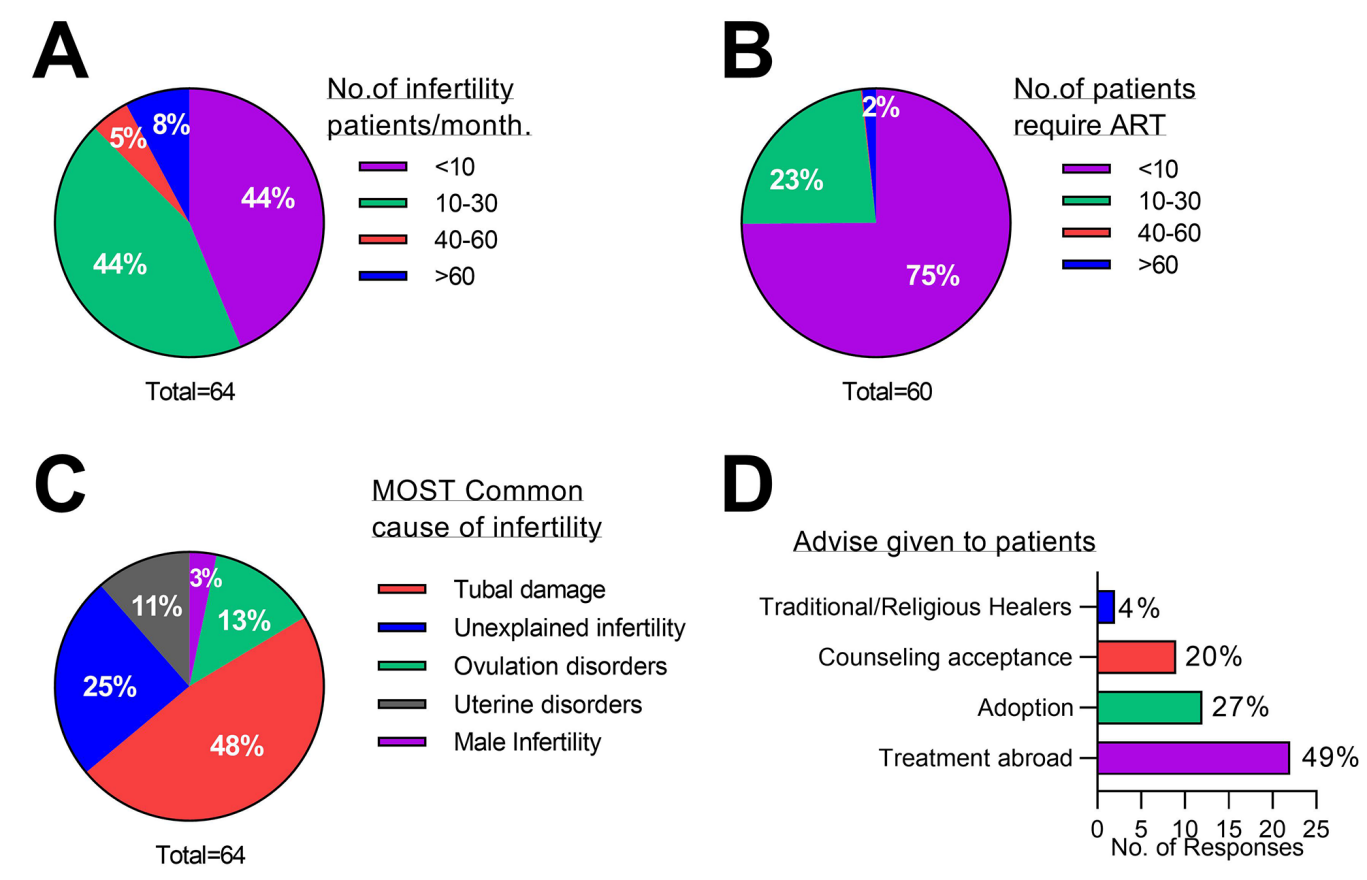
Infertility patients in The Gambia. (**A**) Average number of infertility patients seen by staff per month. (**B**) Average number of patients that require ART treatment seen by staff per month. (**C**) The most common perceived causes of infertility in patients seen by staff. (**D**) The most common advice given to patients that require ART by staff

In response to the status of patients with infertility, a large majority of the respondents (56 out 64 medical staff, 88%) estimated to see approximately 0–30 infertile patients every month. Only 5% (n = 3) cited consulting about 40–60 patients a month (Fig. 2A). Among the infertile patients consulted by the facilities, 75% of the respondents (45 out 60 respondents) indicated that they have consulted with less than ten of their patients per month requiring ART treatment, with none reporting more than 30 patients per month (Fig. 2B). Participants indicated that tubal damage was the most frequent perceived cause of infertility (29 out of 64 respondents, 48%) (Fig. 2C). For those who require follow-up care, most advise their patients to seek treatment abroad (n = 22 of 45 respondents, 49%), whereas very few advise traditional/religious healers (n = 2 out of 45 respondents, 4%) (Fig. 2D).

### Knowledge and motivation to support ART care in the Gambia

**Fig. 3:**
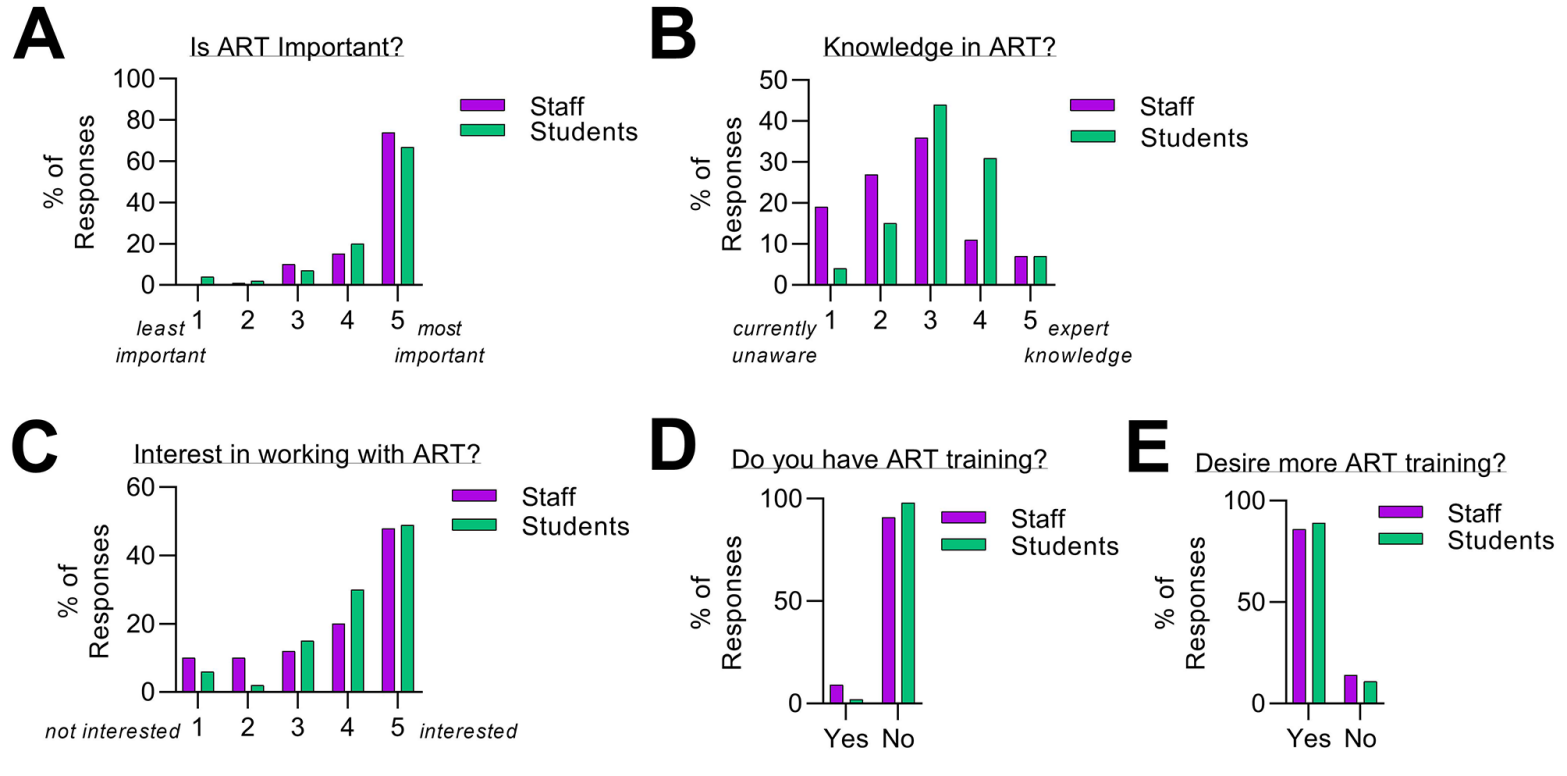
Knowledge and motivation to support infertility care in The Gambia. (**A**) Degree of the importance of ART introduction in the Gambia as viewed by respondents, (1 = least important, 5 = most important). (**B**) Level of confidence in ART knowledge as viewed by respondents (1 = currently unaware, 5 = expert knowledge). (**C**) The respondents level of interest to work with ART services if they become available (1 = not interested, 5 = interested). (**D**) Percentage of formal ART training. (**E**) Percentage of respondents that want additional ART training

Knowledge and motivation questions assessed the perceptions of staff and students in supporting ART services, if these services become available in the country. When questions were analysed between staff and students, there was an overwhelming agreement among the two groups of respondents that it is important to prioritise infertility care and introduce ART (Fig. 3A). Most of the respondents reported their knowledge about ART services as partial (Fig. 3B). However, other items such as “interest in working with ART services” had more variability between the two groups. For example, 68% of staff and 79% of students were interested in working with ART services (responses of 4 or 5, high interest), whereas 20% of staff and 8% of students were not interested (response of 1 or 2, low interest) (Fig. 3C). Further, the majority of both staff and students were not formally trained in ART (91% of staff and 98% of students) (Fig. 3D). Nonetheless, regardless of interest, a majority of all respondents would like to acquire additional formal training in ART in the future (86% of staff and 89% of students) (Fig. 3E).

### Barriers to the introduction of ART in the Gambia

**Fig. 4:**
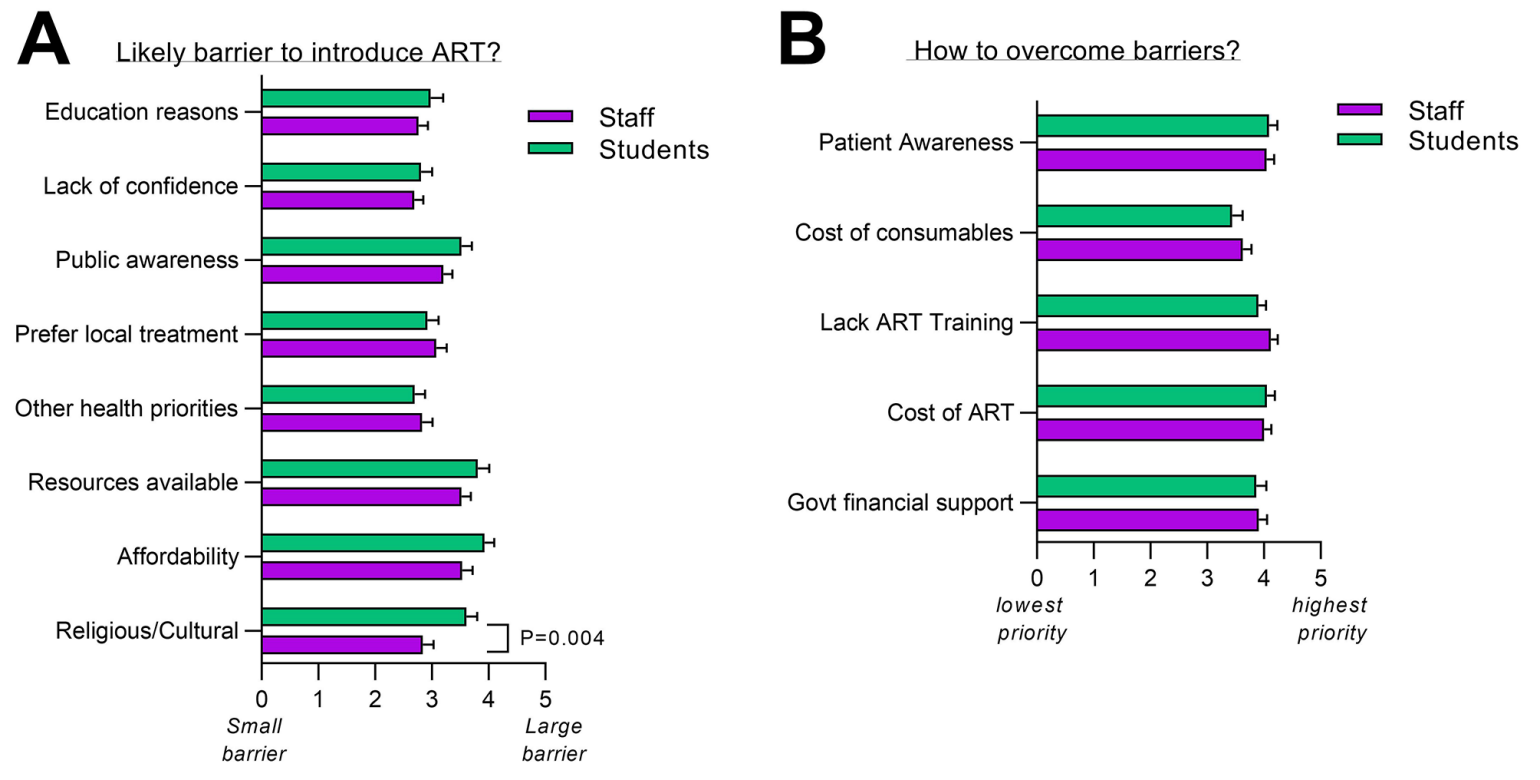
Barriers to the introduction of ART in The Gambia. (**A**) The view of respondents on the most likely barriers to the introduction of ART (1 = small barrier, 5 = large barrier). Statistical significance was determined by multiple t tests between groups. P-value less than 0.05 was considered significant. (**B**) The view of respondents on ways to overcome these barriers (1 = lowest priority, 5 = highest priority). (**A&B**) Data represented as grouped interleaved bar graphs plotted with mean and standard error of mean (SEM)

Respondents were asked to indicate the barriers they perceived might hinder the introduction of ART in The Gambia. Participants answered that affordability, availability of resources, and public awareness were significant obstacles that might prevent the introduction of ART in The Gambia (Fig. 4A). A significant difference was found between staff and students for the perception for religious and cultural reasons as a barrier to ART (p = 0.004). Regarding how to overcome these barriers, both staff and students found patient awareness, government financial support, reduced cost for ART, and additional training in ART techniques equally important (Fig. 4B).

### Financial support for infertility patients

**Fig. 5:**
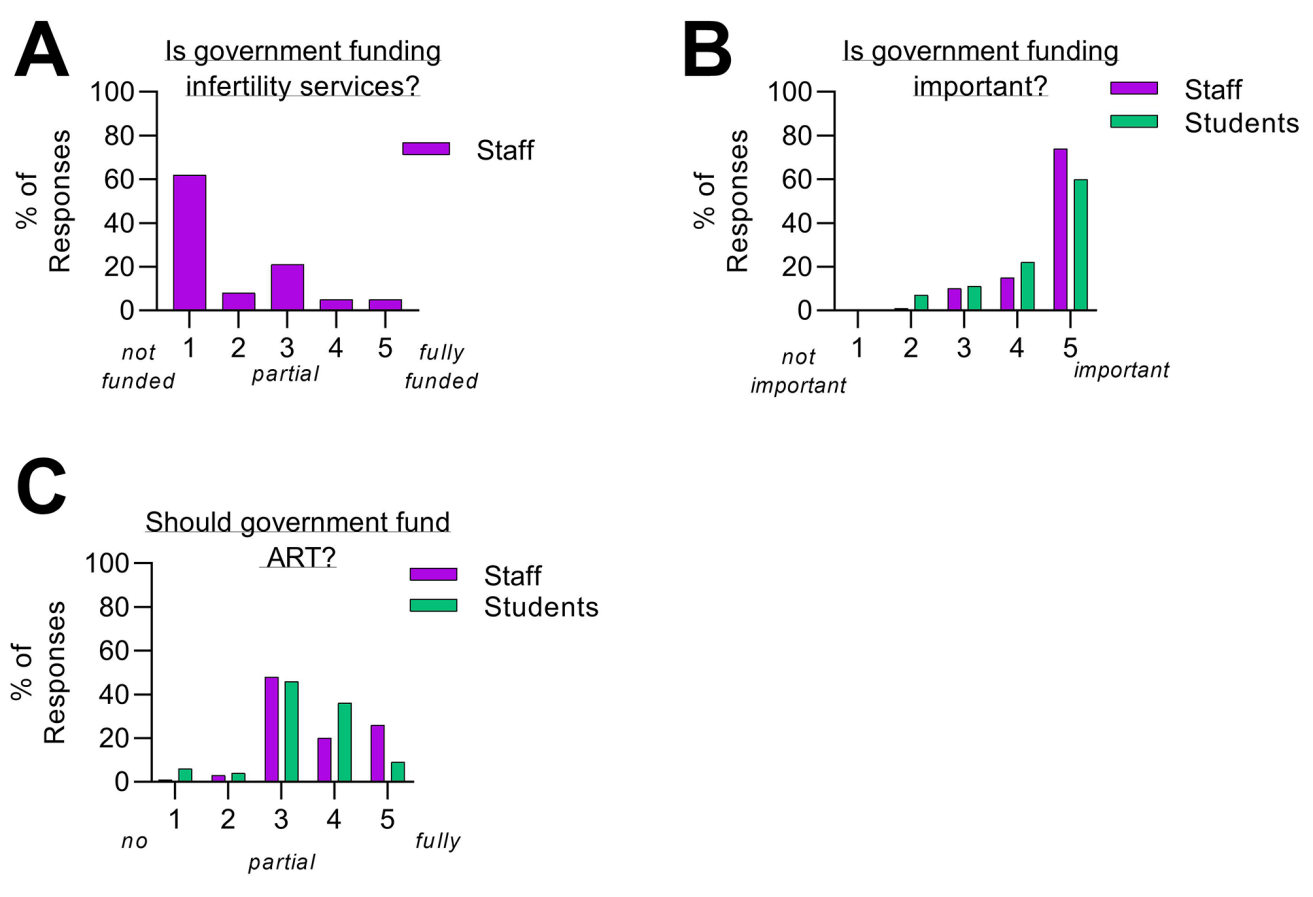
Financial support for infertility patient. (**A**) Staff perception on whether government currently funds infertility services (1 = not funded, 3 = partial funding, 5 = full funding). (**B**) The respondents view on the importance of the government to fund infertility services (1 = not important, 5 = important). (**C**) The respondents view on whether the government should fund ART services, and how much funding should be given (1 = no funding, 3 = partial funding, 5 = fully funded)

Within the questionnaire, the respondents were informed that the cost of an IVF cycle in the UK was almost equal to 260,000 Gambian Dalasi (approx. £4,000). Respondents were then asked about the current funding status for infertility services and whether ART services should be funded by the government in the future. Over half of the respondents (39 out of 63 responses, 62%), indicated that currently, infertility services are not funded by the government (value of 1, not funded at all) (Fig. 5A). Most of the respondents perceived that it is important for the government to fund infertility services in the future (Fig. 5B). However, when respondents were asked if ART services should also be funded by the government, the responses varied with partial funding being the most preferred funding criteria (Fig. 5C).

## Discussion

This survey is the first to gauge the perception of health care providers and medical students regarding the introduction of ART in The Gambia. In general, a majority of participants expressed a positive attitude regarding a potential introduction of ART in the Gambian health system. In addition, the survey revealed barriers in affordability and availability of infertility services as well as an inadequate number of trained infertility care providers in the current and future health workforce. Overall, this study has informed what considerations should be explored prior to introducing ART services in The Gambia.

We found variations in the reported infrastructure and treatments/services provided for infertility among Gambian hospitals. Most of the respondents expressed a gap in the distribution of infertility services between urban and rural areas, with a majority of services reportedly available in the urban areas. Respondents noted that urban hospitals (e.g. EFSTH, Kanifing, Brikama and Serrekunda) have essential infrastructure and do perform investigations required for the provision of fertility care, mainly screening and diagnostic testing. This is in line with Afferri et al. (2022) recent facility-based survey [17]. This is not the case however for a majority of the hospitals in rural areas (e.g. Soma, Bwiam, and Koina) that are lacking in investigations, such as semen analysis, necessary for male factor infertility diagnosis. Unlike infertility investigations and facilities, infertility treatment options were said to be limited for both the urban and rural hospitals in the country, corroborating Afferri et al. (2022) [17]. Among the urban clinics, respondents noted that the EFSTH was the only hospital that offers ovulation induction, IUI and tubal surgery, although this is in contrast to the survey findings [17], and it therefore remains unclear if IUI is available in the Gambia public sector or not. Whereas the rest offer only ovulation induction, excluding Bwiam, MRCG, Gambia Armed Forces clinic and SOS clinic that do not offer any form of infertility treatments. However, as respondents’ identities were blinded with some clinics providing few responses, further investigation is required to corroborate and validate these findings and to provide additional details. Validation of these results with qualitative research (semi-structured interviews and direct observations) is one approach to doing so.

Because of the uneven distribution of infertility services, patients who require fertility care are referred to urban facilities. This is challenging in the rural context due to poverty, remoteness and a lack of transportation. Poor transportation challenges are very common in rural Africa. A study by Atuoye et al. (2015) in Ghana [20] and Northern Nigeria [21] showed rural travel as an issue. The major problem of limited access to medical care, especially obstetrics and gynecology, resulted in patients seeking care with local traditional healers. Ultimately, any delay receiving required (biomedical) treatment may significantly affect the achievement of the treatment outcomes. To improve general fertility care nationwide, it is important to decentralize healthcare staff and services for the benefit of those living in the rural area of the country. However, EFSTH may serve as a first reference to begin introducing ART services in The Gambia.

Overall, respondents understood and highly valued the importance of infertility care and a majority of the staff stated that it is important for the government to fund these services. However, when it comes to the government investing in ART services, this study found that a majority of respondents believe this should be partially funded. Generous public financing schemes towards IVF may show better access to ART services, yet public-private partnerships may be better suited for settings such as The Gambia. While in high-income countries such as Belgium for example, there has been a reimbursement policy for public ART services since 2005 [22] and in 2016 they reported free universal access to ART services for the general public [7], in Africa, only few countries such as Egypt and South Africa, have integrated ART services in their public healthcare sector through government support [22]. Further, South Africa, Uganda and Nigeria have introduced affordable charges of about 200 US dollars per cycle in the last decade [7, 8]. It is therefore not surprising that these countries account for highest number of IVF cycles performed in SSA [23]. For instance, Ombelet (2019) [7] reported South Africa was among the top performers across SSA in terms of the number of cycles, accounting for 4,995 cycles of the total 20,000 cycles performed across the continent in 2016. Further, the high cost of IVF in LMIC is one of the major barriers in access to treatment of infertility [8, 23]. Thus, reimbursement (partial or total) for ART services from the government will be an additional support required for successful inclusion of ART in The Gambia. How to best implement this support remains undefined.

A majority of the respondents had some basic knowledge and understanding about general infertility care. A study conducted in The Gambia by Sisawo and colleagues in 2017 [24] showed that the Gambian healthcare system staff fall under different categories: (1) medical doctors; (2) nurses - formally trained; and (3) basic nurses – no formal training in nursing, also called community nurses. Basic nurses are trained on the job, and only provide preventive care and treatment of minor complaints – not infertility services. They are also usually the primary health care providers in the rural areas. Sisawo reported that about 75% of the staff composition of the healthcare work force in The Gambia are nurses [24]. There is no available data about the percentage of basic nurses providing care throughout the country. Given that 40% of the staff surveyed here were not formally trained, most of these would likely be basic nurses. However, 98% of the medical students surveyed received formal training in providing infertility care. Information regarding the training of nursing students in infertility care is currently unavailable. Considering that medical students will provide health care within 3 years, it’s important to capture their education level on infertility. Results from this study showed the need for specialized training on infertility for all medical and nursing students, regardless of field of specialization. Contrary to general infertility training, a large majority of the respondents are neither trained nor have knowledge in ART services. As seen in a recent study in Kenya (2015), having inadequately trained embryologists meant that clinics had no choice but to hire expatriates, which meant that fertility care centers charged higher rates per cycle. In all, a lack of expertise in the field of ART is a major obstacle to setting up and running IVF clinics in Africa [25, 26]. To implement and sustain an assisted conception unit soon, the Gambian government needs to seek opportunities to fund specialized training of interested Gambian doctors through collaborations with international universities and institutes in order to acquire skills such as IVF specialist and embryologists. One such programme is currently in development through the Fertility Care in the Global South Network.

### Limitations

This study may be over-representative of staff perspectives at the major hospital centres (i.e. EFSTH, Kanifing, Brikama), compared to the minor hospitals, as few responses were provided from minor hospitals. However, this is likely also reflective of fewer staff involved in infertility located in these rural areas. Furthermore, the opinions provided are not representative of the general public as only healthcare providers and students were surveyed. As highlighted by Dierickx et al. (2021), the biomedical knowledge of people living with infertility in The Gambia is limited [27], and likely dependent on access to education, which is very different to that of the healthcare professionals. Given the overrepresentation of data of the population aged 18–25 and females, this could mean that there is an overrepresentation of people in childbearing age who are interested in fertility and their responses in the survey may be biased. However, this was not deemed as a key limitation as the main focus of this survey is the perception of health care providers and medical students regarding the introduction of ART in The Gambia. Also, when surveying staff for the frequency of infertility/ART patients, the questionnaire did not capture responses for 31–39 patients per month, as a result of a technical error during survey design which omitted this option; listing only < 10, 10–30, 40–60, and > 60 patients per month. This may have slightly biased the results for this particular question. Next, the introductory note stated that in previously published information, tubal damage and male factor infertility were prevalent in The Gambia. This statement may have also somewhat biased staff responses – although this is unlikely given that male factor infertility was the least common option chosen by the participants. Lastly, data for this study was entirely dependent on information provided by the participants. Therefore, health system readiness and clinical evidence regarding the causes of infertility, along with a validation of the healthcare infrastructure, couldn’t be ascertained. In the future, in-person site visits and one-to-one interviews with healthcare staff and Ministry of Health representatives are recommended.

## Conclusion

Infertility is a major concern in The Gambia, a pro-natalist society where procreation in marriage is deemed extremely important. This survey revealed positive attitudes towards the introduction of ART in The Gambia by current and future health care providers as well as their interest to incorporate ART in their professional capacity, especially among the younger generation. However, there is significant lack of formally trained personnel providing infertility care and thus, a major need for trained IVF specialists or embryologists. To provide high quality infertility care services including ART, the government may need to invest in training Gambians who are interested in reproductive health, including embryologist, to ensure a successful setting up and sustainability of an assisted conception unit in The Gambia. This survey also identified limited infrastructure capability and infertility treatment options as a major issue countrywide, especially in the rural areas. Here, the majority of the hospitals lack basic infertility care services, suggesting the engagement of the government to support the provision of basic infertility in the rural areas to all patients is important.

## Electronic supplementary material

Below is the link to the electronic supplementary material.

Supplementary Material 1

## Data Availability

Survey responses are available through the Open Science Framework (10.17605/OSF.IO/ARWBS). The analyzed datasets are available from the corresponding author upon reasonable request.

## List of abbreviations

ART: Assisted Reproduction Technology
EFSTH: Edward Francis Small Teaching Hospital
GARD: Association of Resident Doctors The Gambia
HIV: Human immunodeficiency virus
IVF: In Vitro Fertilization
ICSI: Intracytoplasmic sperm injection
IUI: Intrauterine insemination
LMIC: Low and Middle-Income Countries
SEM: Standard error of mean
SSA: sub-Saharan Africa
UTG: University of The Gambia

## References

[1] Thoma M, Fledderjohann J, Cox C, Adageba RK. Biological and social aspects of human infertility: a global perspective. In: *Oxford Research Encyclopedia of Global Public Health* edn.; 2021.

[2] Ericksen K, Brunette T (1996). Patterns and predictors of infertility among african women: a cross-national survey of twenty-seven nations. Soc Sci Med.

[3] Geelhoed DW, Nayembil D, Asare K, van Schagen JH, van Roosmalen J (2002). Infertility in rural Ghana. Int J Gynaecol Obstet.

[4] Akalewold M, Yohannes GW, Abdo ZA, Hailu Y, Negesse A (2022). Magnitude of infertility and associated factors among women attending selected public hospitals in Addis Ababa, Ethiopia: a cross-sectional study. BMC Womens Health.

[5] Sundby J, Mboge R, Sonko S (1998). Infertility in the Gambia: frequency and health care seeking. Soc Sci Med.

[6] Mascarenhas MN, Flaxman SR, Boerma T, Vanderpoel S, Stevens GA (2012). National, Regional, and global Trends in Infertility Prevalence since 1990: a systematic analysis of 277 health surveys. PLoS Med.

[7] Ombelet W, Onofre J (2019). IVF in Africa: what is it all about?. Facts Views Vis Obgyn.

[8] Inhorn MC, Patrizio P (2015). Infertility around the globe: new thinking on gender, reproductive technologies and global movements in the 21st century. Hum Reprod Update.

[9] Ombelet W, Campo R (2007). Affordable IVF for developing countries. Reprod Biomed Online.

[10] Dhont N, van de Wijgert J, Coene G, Gasarabwe A, Temmerman M (2011). ’Mama and papa nothing’: living with infertility among an urban population in Kigali, Rwanda. Hum Reprod.

[11] Chiware TM, Vermeulen N, Blondeel K, Farquharson R, Kiarie J, Lundin K, Matsaseng TC, Ombelet W, Toskin I (2021). IVF and other ART in low- and middle-income countries: a systematic landscape analysis. Hum Reprod Update.

[12] van Balen F, Gerrits T (2001). Quality of infertility care in poor-resource areas and the introduction of new reproductive technologies. Hum Reprod.

[13] Bahamondes L, Makuch MY (2014). Infertility care and the introduction of new reproductive technologies in poor resource settings. Reproductive Biology and Endocrinology.

[14] Klouman E, Masenga EJ, Klepp KI, Sam NE, Nkya W, Nkya C (1997). HIV and reproductive tract infections in a total village population in rural Kilimanjaro, Tanzania: women at increased risk. J Acquir Immune Defic Syndr Hum Retrovirol.

[15] Favot I, Ngalula J, Mgalla Z, Klokke AH, Gumodoka B, Boerma JT (1997). HIV infection and sexual behaviour among women with infertility in Tanzania: a hospital-based study. Int J Epidemiol.

[16] Lancaster P, de Mouzon J. Global ART Surveillance:The International Committee Monitoring Assisted Reproductive Technologies (ICMART). 10.1016/j.fertnstert.2006.04.018 16762350

[17] Afferri A, Allen H, Dierickx S, Bittaye M, Marena M, Pacey A, Balen J (2022). Availability of services for the diagnosis and treatment of infertility in the Gambia`s public and private health facilities: a cross-sectional survey. BMC Health Serv Res.

[18] Anyanwu M, Idoko P (2017). Prevalence of infertility at the gambian Teaching Hospital. Womens Heaith Gynecol.

[19] Joshi A, Kale S, Chandel S, Pal DK (2015). Likert scale: explored and explained. Br J Appl Sci Technol.

[20] Atuoye KN, Dixon J, Rishworth A, Galaa SZ, Boamah SA, Luginaah I (2015). Can she make it? Transportation barriers to accessing maternal and child health care services in rural Ghana. BMC Health Serv Res.

[21] Essien E, Ifenne D, Sabitu K, Musa A, Alti-Mu’azu M, Adidu V, Golji N, Mukaddas M (1997). Community loan funds and transport services for obstetric emergencies in northern Nigeria. Int J Gynaecol Obstet.

[22] Ombelet W (2007). Access to assisted reproduction services and infertility treatment in Belgium in the context of the european countries. Pharmaceuticals Policy and Law.

[23] Dyer S, Archary P, Potgieter L, Smit I, Ashiru O, Bell EG (2020). Assisted reproductive technology in Africa: a 5-year trend analysis from the African Network and Registry for ART. Reprod Biomed Online.

[24] Sisawo EJ, Ouédraogo SYYA, Huang S-L (2017). Workplace violence against nurses in the Gambia: mixed methods design. BMC Health Serv Res.

[25] Adageba RK, Maya ET, Annan JJ, Damalie FJ (2015). Setting up and running a successful IVF program in Africa: prospects and Challenges. J Obstet Gynaecol India.

[26] Ombelet W, Goossens J (2016). The walking Egg Project: how to start a TWE centre?. Facts Views Vis Obgyn.

[27] Dierickx S, Balen J, Longman C, Rahbari L, Clarke E, Jarju B, Coene G (2019). ’we are always desperate and will try anything to conceive’: the convoluted and dynamic process of health seeking among women with infertility in the West Coast Region of the Gambia. PLoS ONE.

