# Supplementary material for: Introducing assisted reproductive technologies in The Gambia, a survey on the perspectives of Gambian healthcare professionals and medical students
Source: BMC Health Serv Res. 2023 Feb 28;23:203. doi: 10.1186/s12913-023-09171-7 (PMC9979844; doi:10.1186/s12913-023-09171-7)
Supplement: Supplementary file 1 — Supplementary Material 1 [file 12913_2023_9171_MOESM1_ESM.docx]

**SUPPLEMENTAL INFORMATION**

**Perspectives on the introduction of Assisted Reproductive Technologies in The Gambia by healthcare professionals and medical students.**

Haddy Bittaye^1#^; Jason P. Mooney^2^; Anna Afferri^3^; Julie Balen^3^; Vanessa Kay^4^

^1^ Wellingara, Serekunda, The Gambia

^2^ Institute of Immunology and Infection Research, School of Biological Sciences, University of Edinburgh, Edinburgh, United Kingdom.

^3^ School of Health and Related Research, University of Sheffield, Sheffield, United Kingdom.

^4^ School of Medicine, University of Dundee, Dundee, United Kingdom.

^#^Correspondence address: Haddy Bittaye, Wellingara, Serekunda, The Gambia.

E-mail: bittayehaddy16@gmail.com

**KEY:**

Appendix 1: Ethical approval letters.

Appendix 2: Participant Information Sheet

Appendix 3: Survey questionnaire of staff.

Appendix 4: Survey questionnaire of students.

**Appendix 1: Ethical approval letters.**


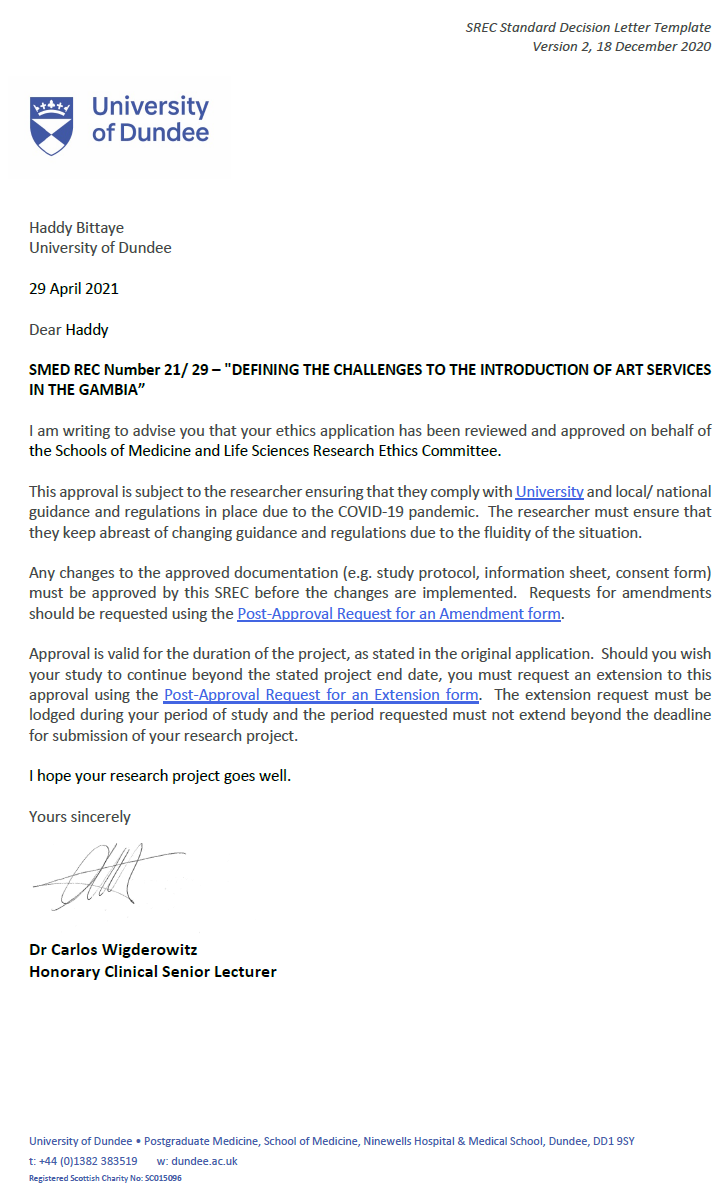


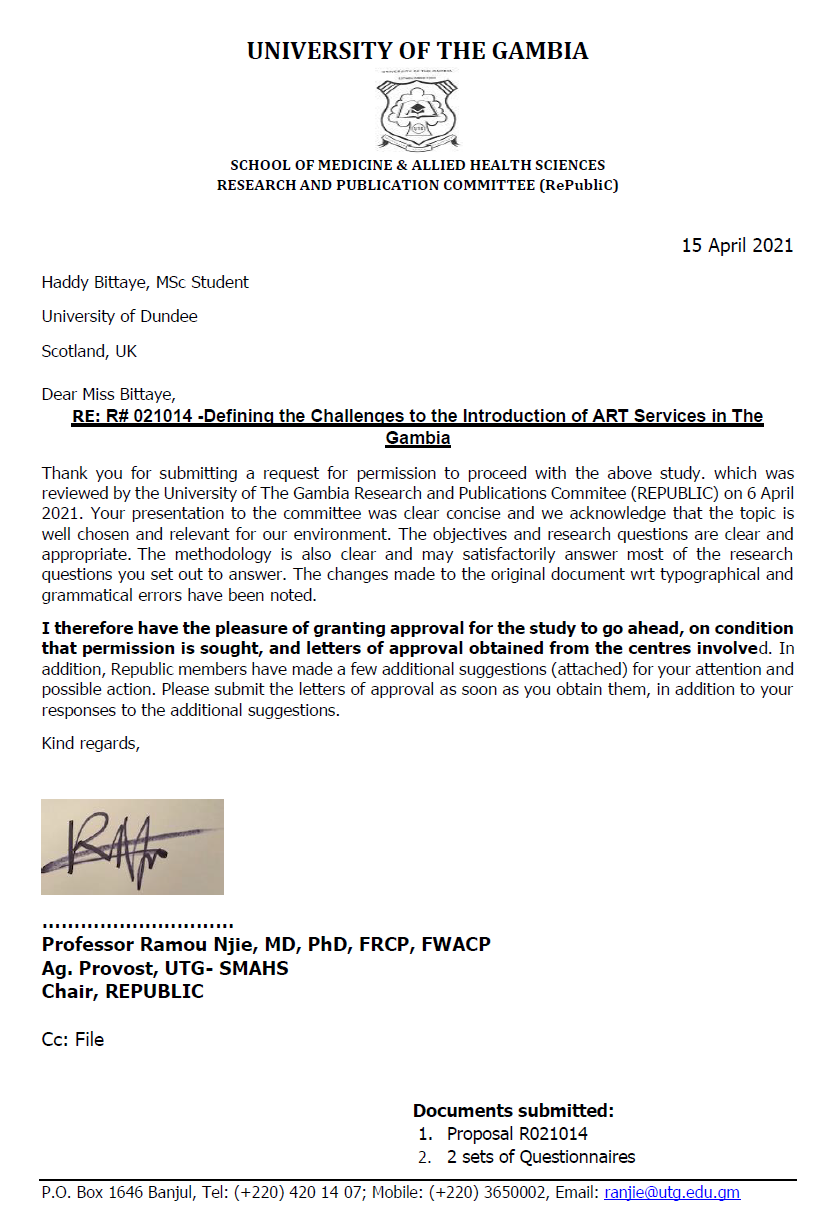


**Appendix 2: Participant Information sheet.**

*
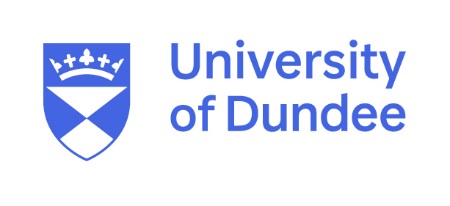
*

*Participant Information Sheet Template*

*Version 1, 8^th^ April 2019*

**Participant Information Sheet for Healthcare professionals and Medical students in The Gambia**

**Project Title: Defining the challenges to the introduction of ART services in The Gambia.**

You are being invited to take part in a research project. Before you make any decision, it is important for you to understand the purpose of the research and what participation will involve. Please read the following information carefully and discuss it with others if you wish. if there is anything that is not clear or if you would like more information please contact the primary researcher, Haddy Bittaye at 170020500@dundee.ac.uk

**Who is conducting the research?**

This Research Project is being conduct by Ms Haddy Bittaye as part of my MSc program.

Haddy Bittaye MSc Student Researcher [170020500@dundee.ac.uk](mailto:170020500@dundee.ac.uk)

Dr. Vanessa Kay Supervisor v.kay@dundee.ac.uk

**Who is funding the research?**

This research is not funded

**What is the purpose of the research?**

The Gambia is one country in West Africa which currently lack any IVF facilities despite the number of couples experiencing fertility challenges. The most common causes of infertility in The Gambia is tubal factor (17.4%) and male factor (8.9%) infertility. Assisted reproduction technologies which are the most effective treatment options for these causes of infertility is currently not available in the country.

The purpose of this research is to assess the barriers to the introduction of Assisted Reproduction Services in The Gambia. This study seeks to:1) assess the current infertility care services and infrastructure; 2) assess the motivation of staff to support ART services ;3) highlight the potential barriers in the introduction of assisted reproduction technologies. In order to evaluate the unmet IVF needs and impact it would have if it were available, this study will survey the current and future health care professional in The Gambia.

**Why have I been invited to take part?**

You have been invited to take part because of your exposure to infertility management and understanding of the subject.

**Do I have to take part?**

No. You can choose to not take part in this study. If you decided to take part in this study you will be provided with the electronic copy of this information sheet to keep and consent will be required at the end of the survey questionnaire. You are still free to withdraw at any time and without giving a reason after deciding to take part. You may opt out of the study before the 30^th^ June 2021 because after data analysis is complete participant may not be able withdraw.

**What will happen if I take part?**

Participating in this study will involve a voluntary completion of survey questionnaire, which will take about 8-10 minutes in duration. This study will be remotely conducted in The Gambia using google forms. Because the study is conducted across two countries ethical approval would be sought through University of The Gambia ethics committee. Questions are divided into five sections and related to information about you, current practice, knowledge on ART, training and funding. You will be able to skip any questions that you do not wish to answer.

**Are there any risks in taking part?**

**Please note**: The intent of this research is to assess the current services, staff motivation and challenges. We won’t be covering any sensitive or embarrassing issue. This means there is no known risk to participants in the study.

**What are the possible benefits of taking part?**

We cannot guarantee that taking part will benefit you directly. By taking part in this study you will help us to understand what to consider when bringing ART services in The Gambia. Results from the study will highlight the potential barriers in the introduction of assisted reproduction services in The Gambia. Overall to better understand of how to improve infertility health care in The Gambia.

**Will my taking part in this project be kept confidential?**

The questionnaires are confidential and participant responses will be anonymous. Email addresses will be kept confidential during the course of this study and the data will only be accessed by the lead researcher and her supervisors. This will include storing computer files on password protected computers. Your responses to the questionnaires will not be linked to you personally.

The responses you provide will be used in the researcher’s masters project thesis with no personal data published. Direct quotes from the open questions may be used but will remain completely anonymous. All the information collected from you will be destroyed after the project, excluding the printed facts.

**What will happen to the information I provide?**

Upon completion of the survey, participant responses will be loaded into the SPPS version 27 software for analysis. Data will be anonymously represented in the research thesis and presentations. No legal names or data from participants who decided to opt out from the study before the 30^th^ June 2021 will be used in the downstream analysis and report write up. Participant responses from the survey will be seen by the researcher and research supervisors. No persons will be identified in any report or presentation. The full research thesis and any subsequent graphs, charts or tables will be accessible by request.

**Data Protection**

To understand the background of participants answering these questionnaires you will be asked personal information about your age occupation and religious belief.

The University asserts that it lawful for it to process your personal data in this project as the processing is necessary for the performance of a task carried out in the public interest or in the exercise of official authority vested in the controller.

The University asserts that is lawful for it to process special categories of your personal data in this project as the processing is necessary for archiving purposes in the public interest, scientific or historical research purposes or statistical purposes in accordance with Article 89(1) of the General Data Protection Regulation *[see Appendix 1 for guidance on the requirements for processing special categories of personal data in your project]*.

The University of Dundee is the data controller for the personal and/or special categories of personal data processed in this project *[Where projects are developed in partnership this section should be amended to reflect the relationships between the partners and their roles in respect of the personal data. This will normally be governed by the collaboration/partnership agreement and associated data sharing agreement].*

The University respects your rights and preferences in relation to your data and if you wish to update, access, erase, or limit the use of your information, please let us know by emailing [170020500@dundee.ac.uk](mailto:170020500@dundee.ac.uk). Please note that some of your rights may be limited where personal data is processed for research, but we are happy to discuss that with you. If you wish to complain about the use of your information please contact the University’s Data Protection Officer in the first instance (email: [dataprotection@dundee.ac.uk](mailto:dataprotection@dundee.ac.uk)). You may also wish to contact the Information Commissioner’s Office (<https://ico.org.uk/>).

You can find more information about the ways that personal data is used at the University at: <https://www.dundee.ac.uk/information-governance/dataprotection/>.

**Is there someone else I can complain to?**

If you wish to complain about the way the research has been conducted please contact the Convener of the University Research Ethics Committee (<https://www.dundee.ac.uk/research/ethics/contacts/>).

**Alternative formats**

**Alternative forms of this information sheet will be available for you if required***. Advice on alternative formats can be obtained from* [*Disability Services*](https://www.dundee.ac.uk/disabilityservices/services/alternativeformats/) *(email:* [*altformats@dundee.ac.uk*](mailto:altformats@dundee.ac.uk)*).*

**Appendix 3: Survey questionnaire of staff.**


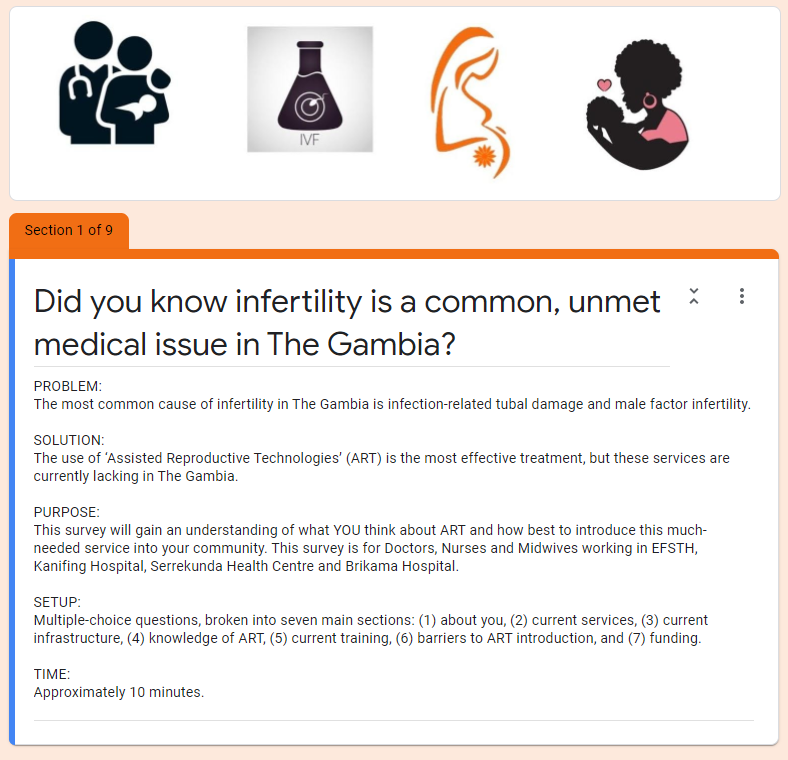


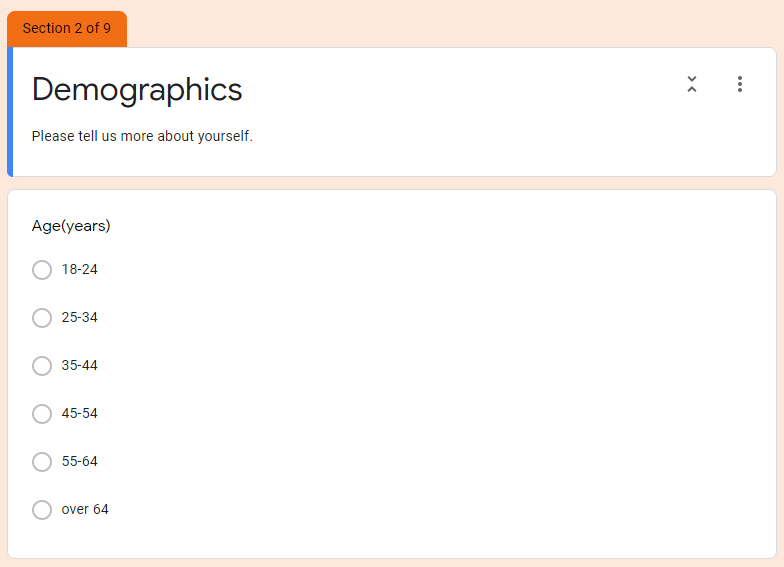


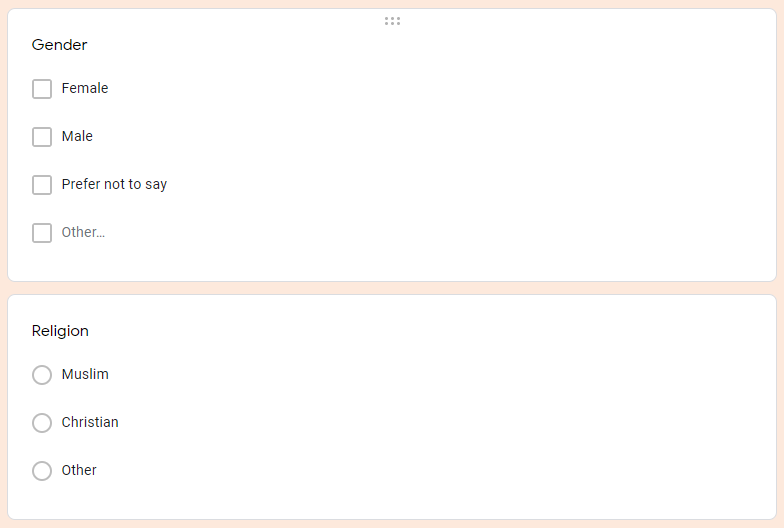


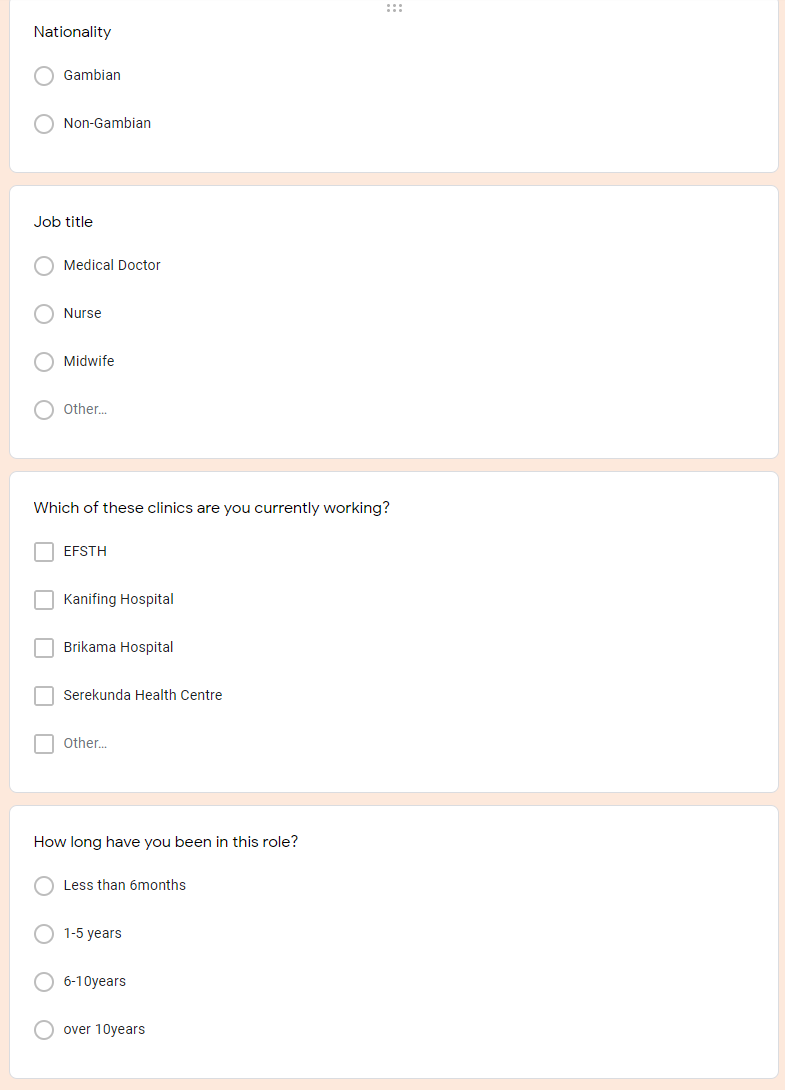


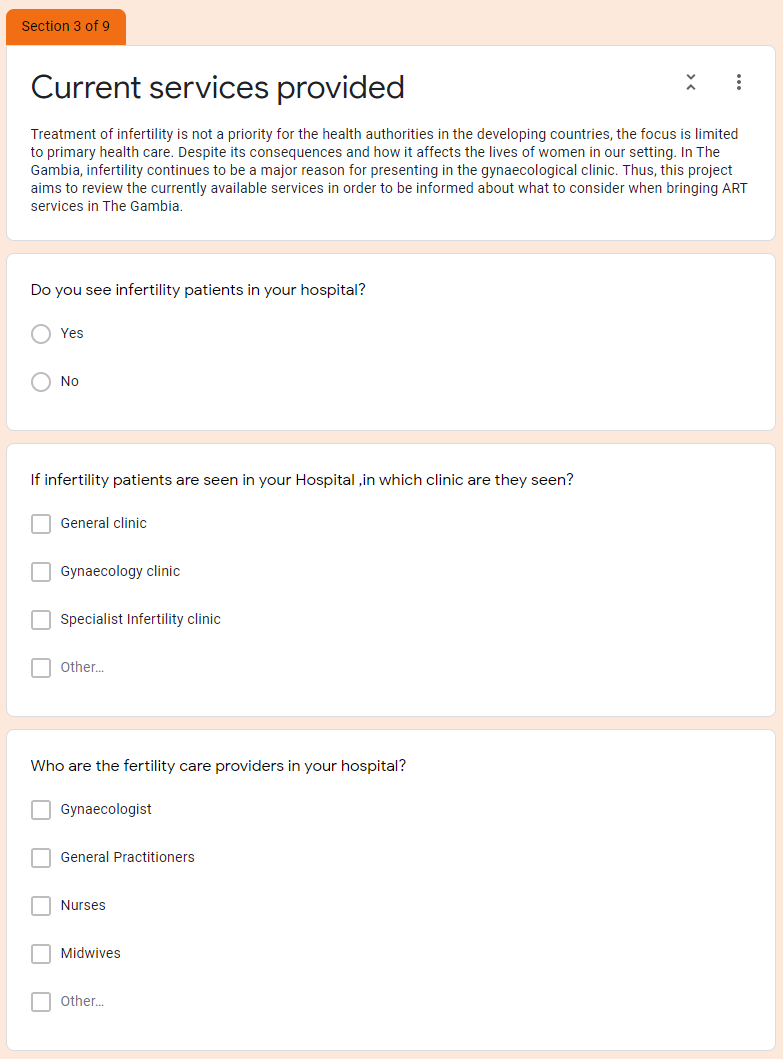


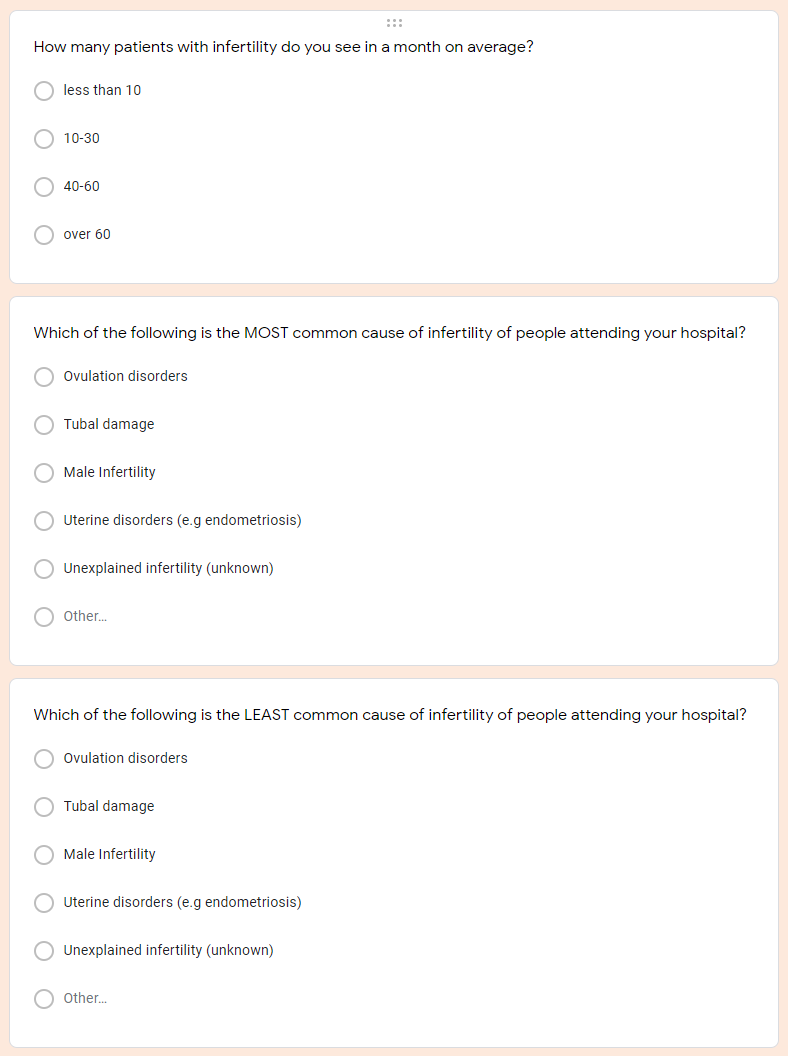


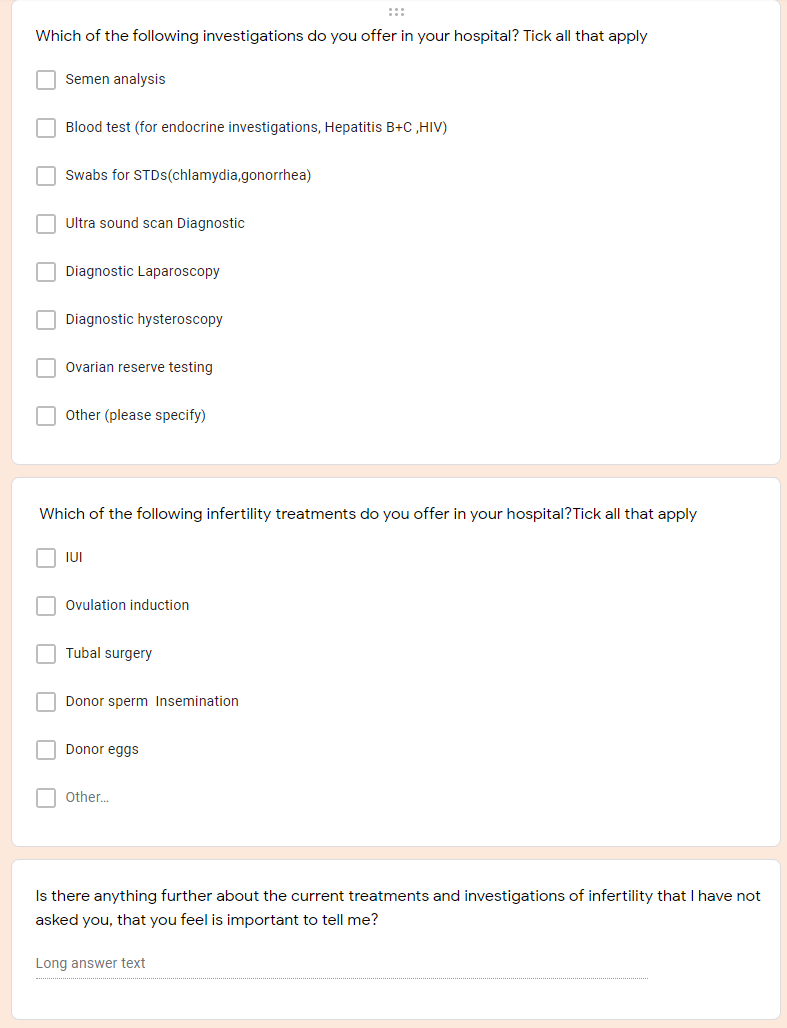


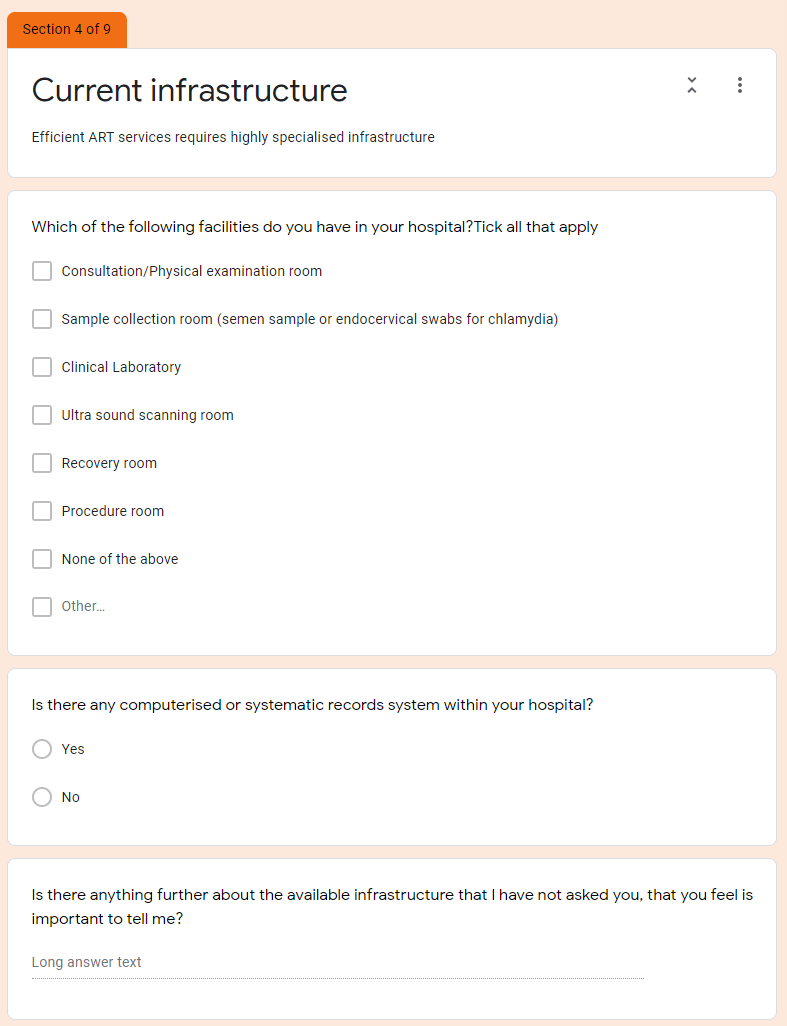


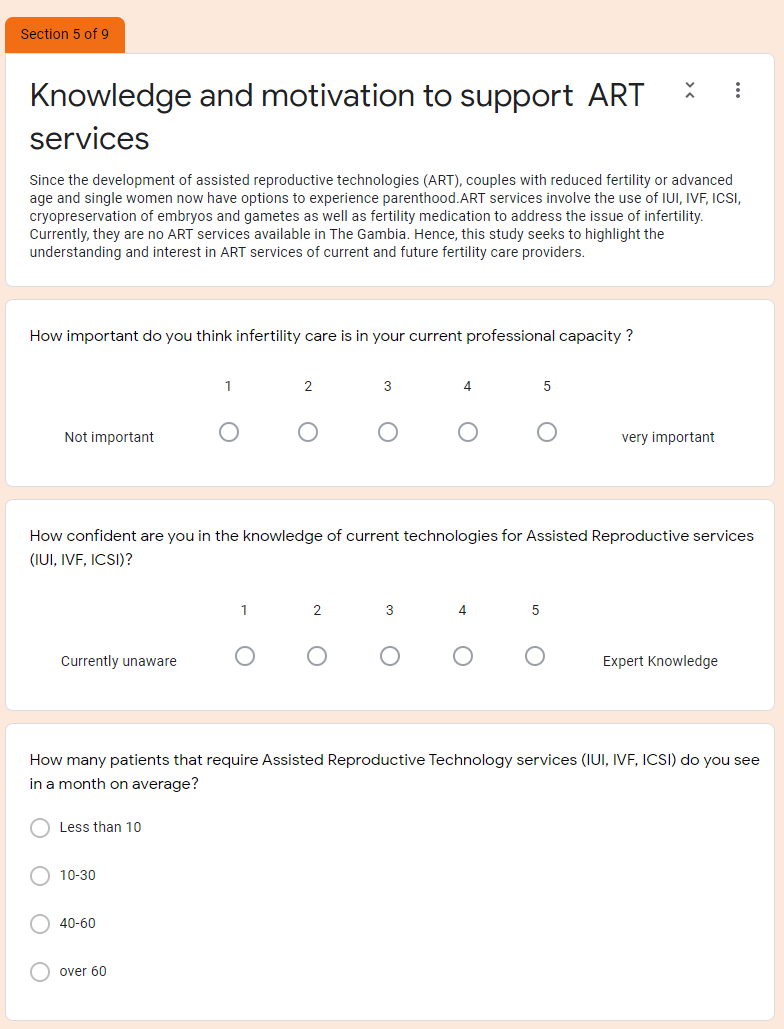


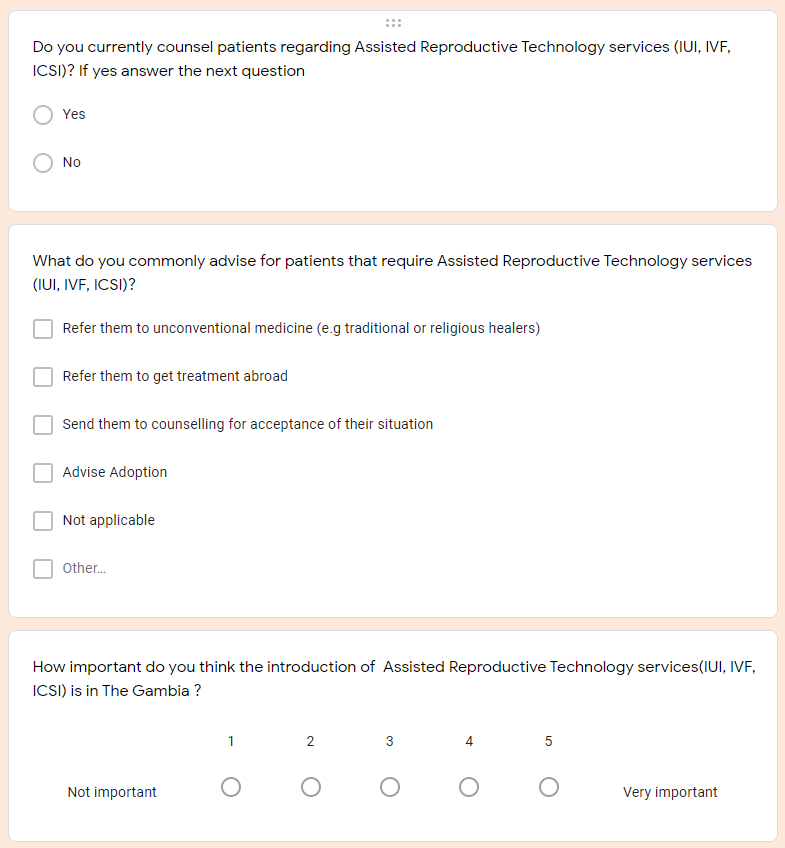


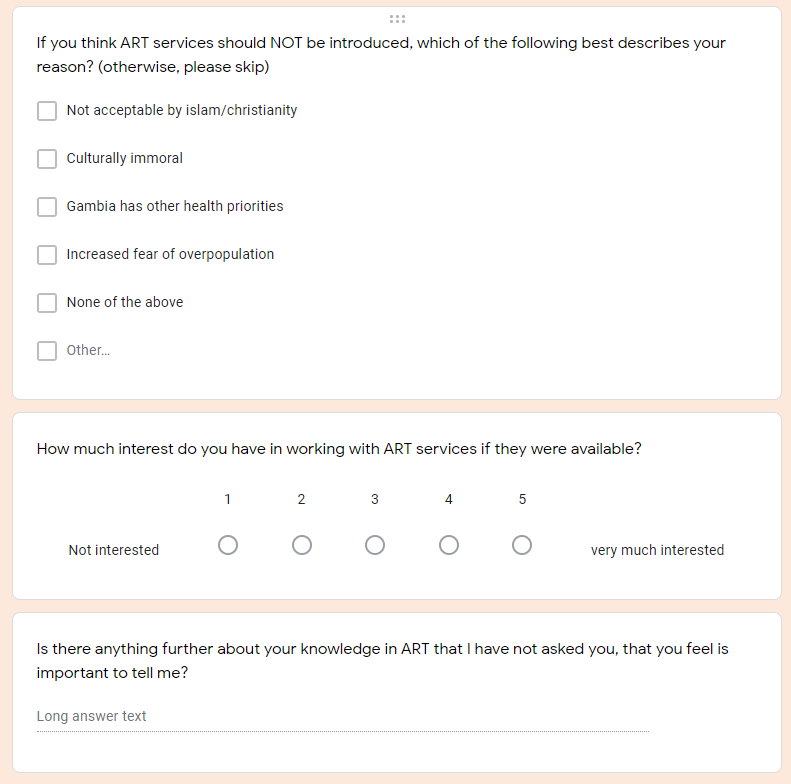


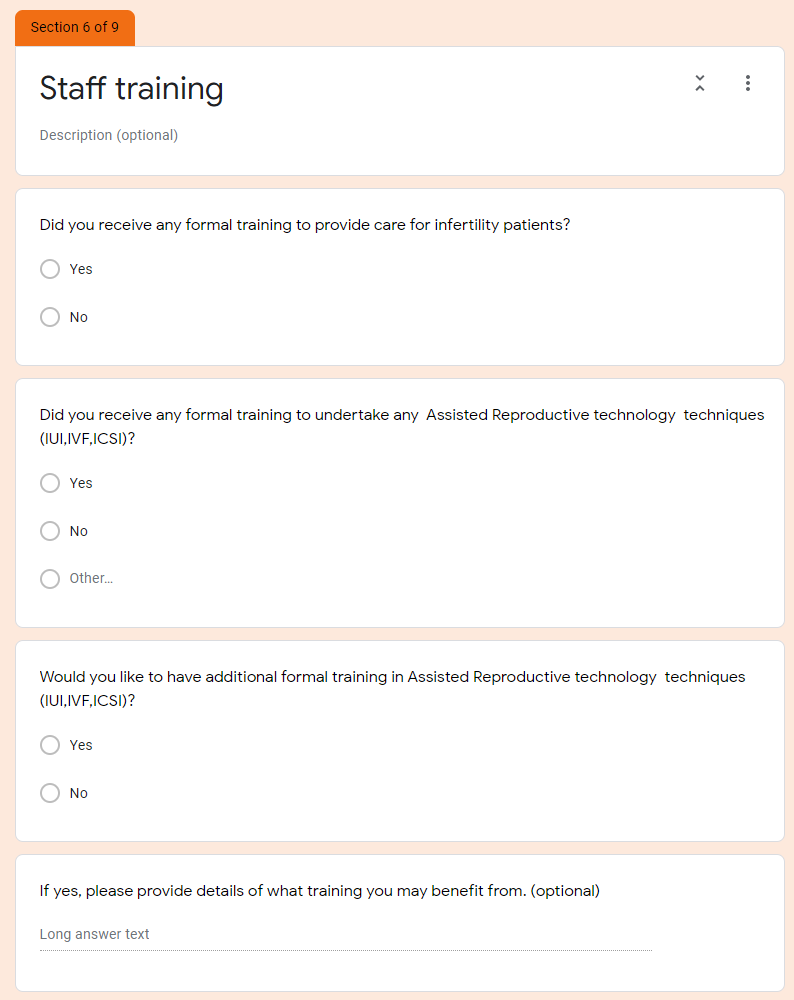


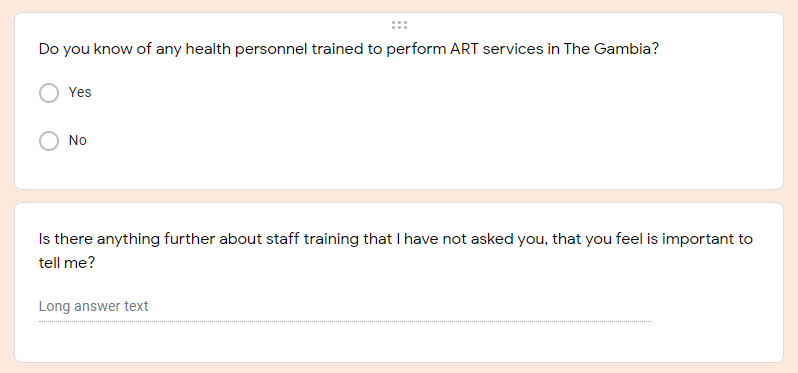


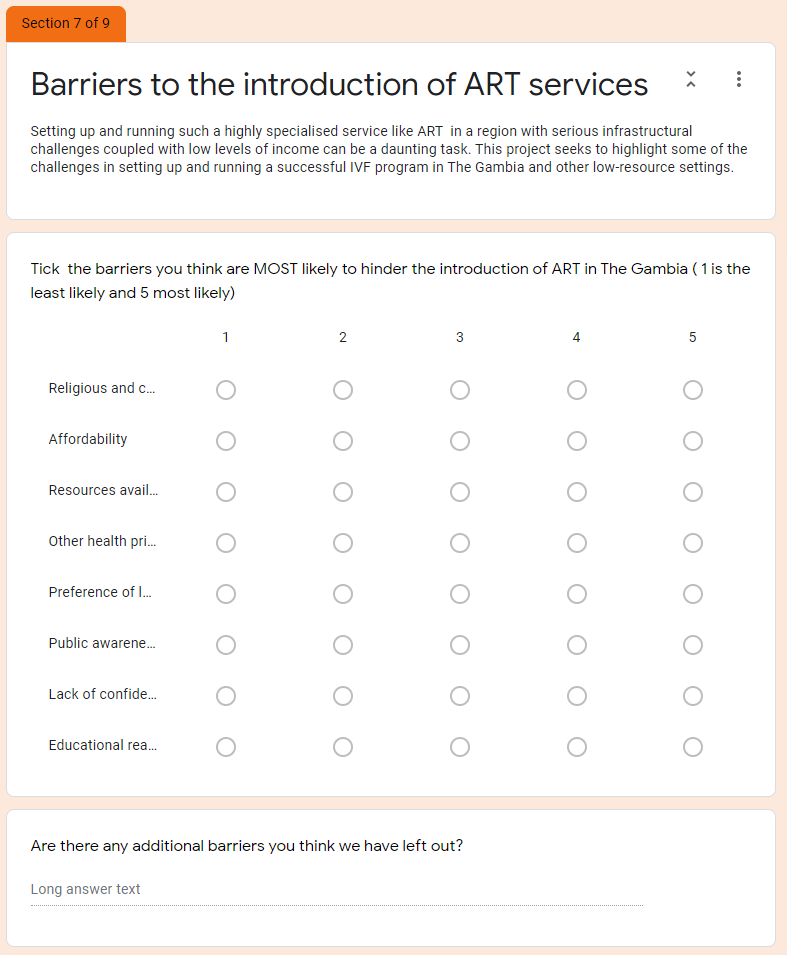


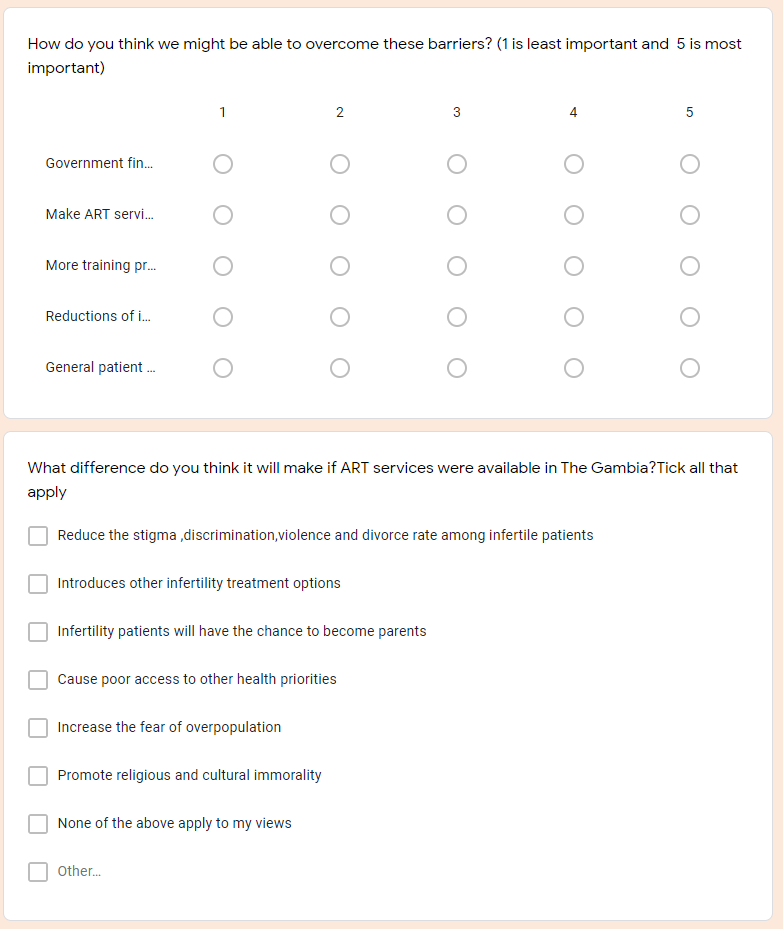


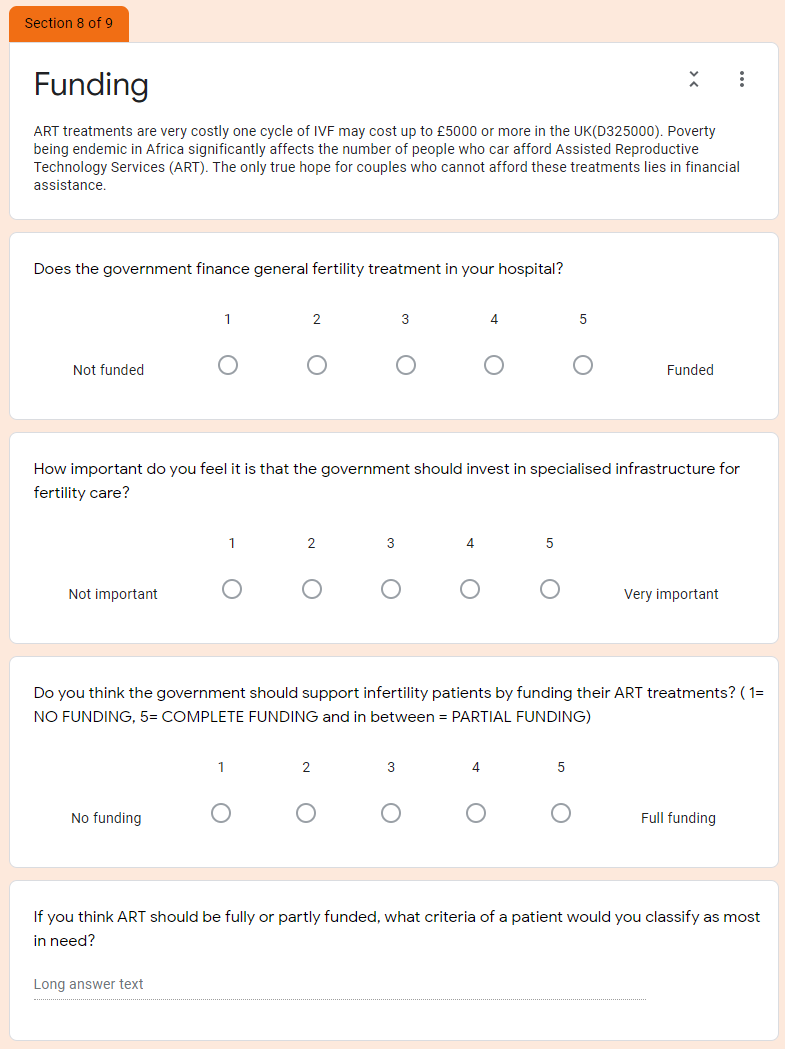


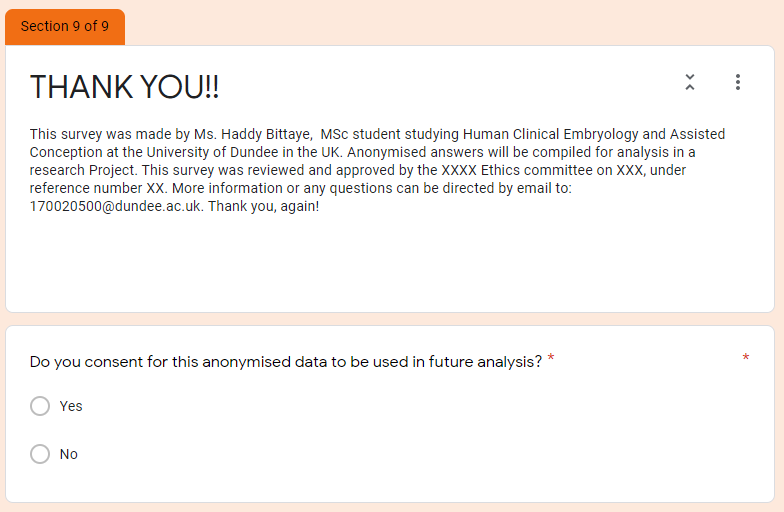


[Forms](https://www.google.com/forms/about/?utm_source=product&utm_medium=forms_logo&utm_campaign=forms)

**Appendix 4: Survey questionnaire of students.**


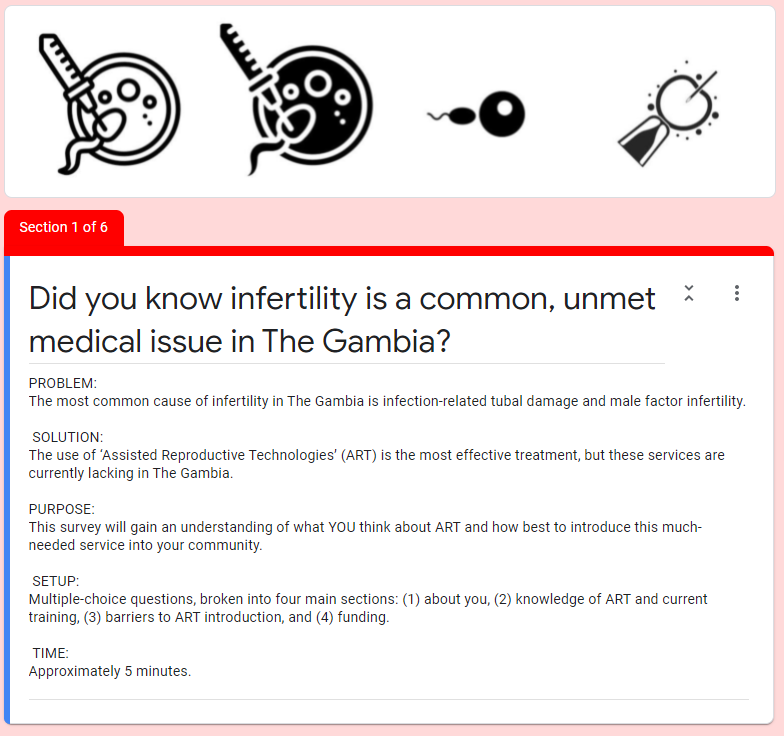


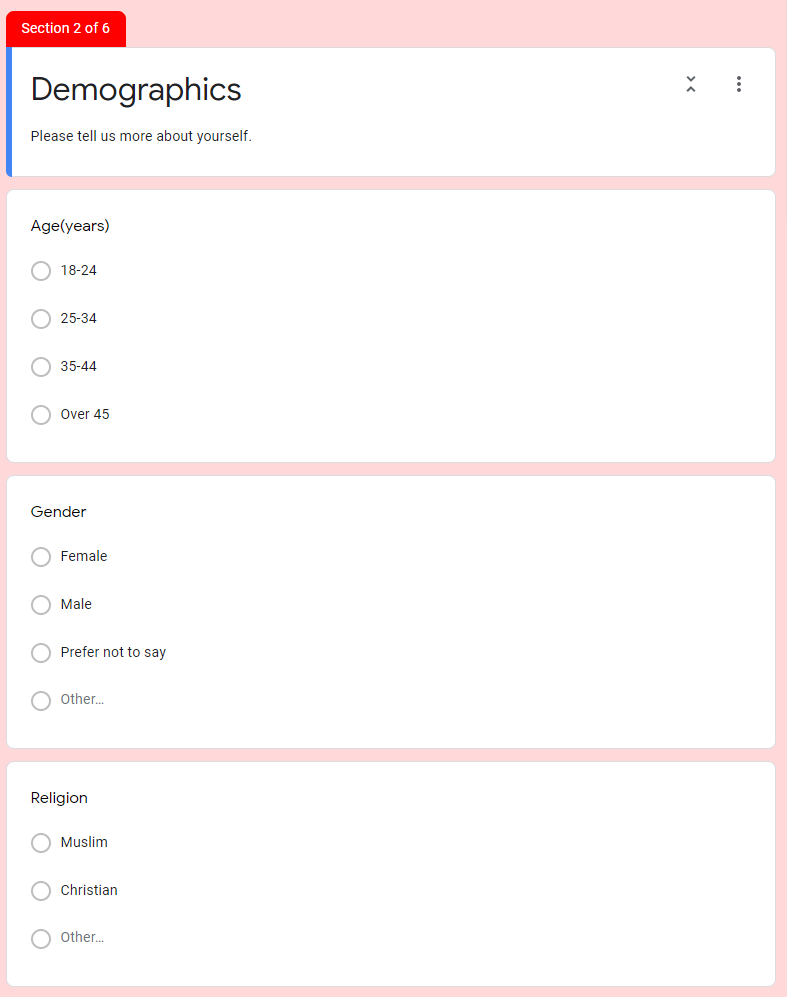


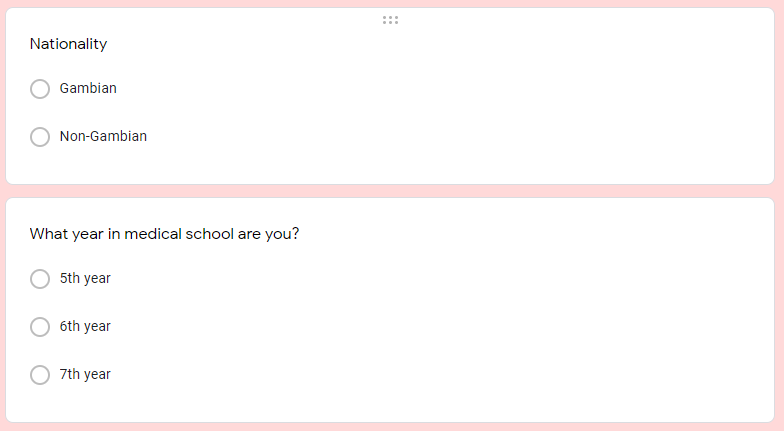


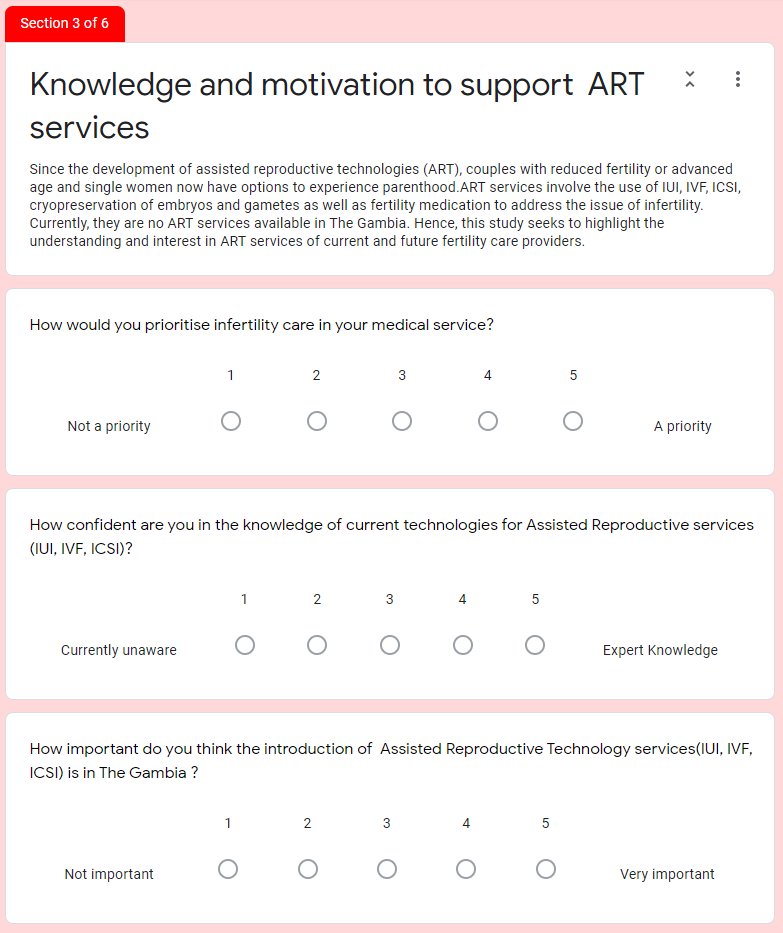


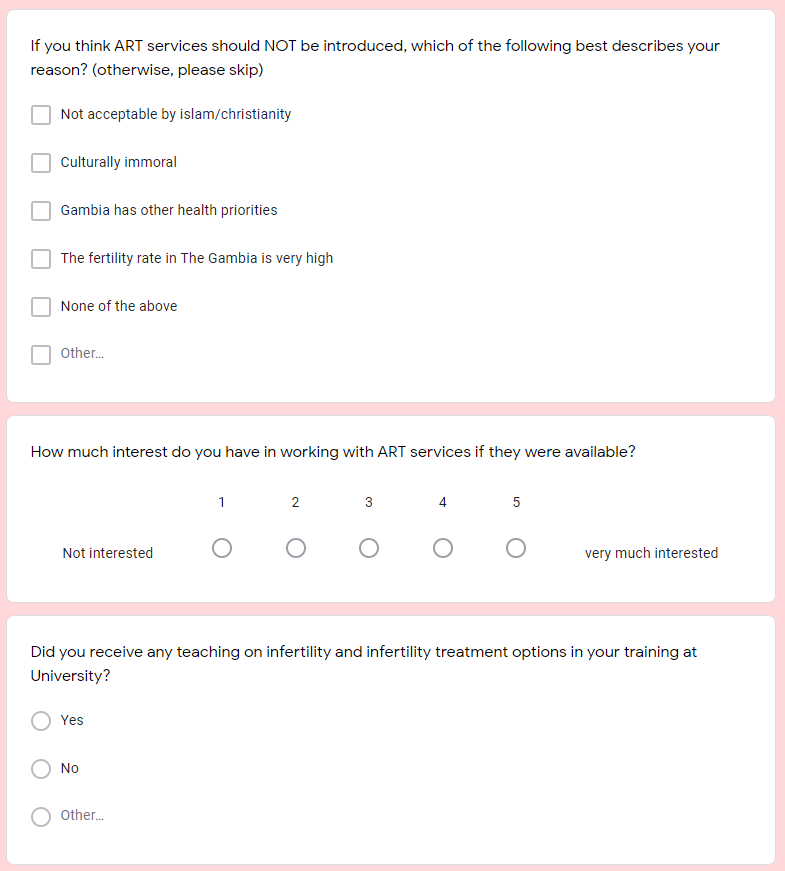


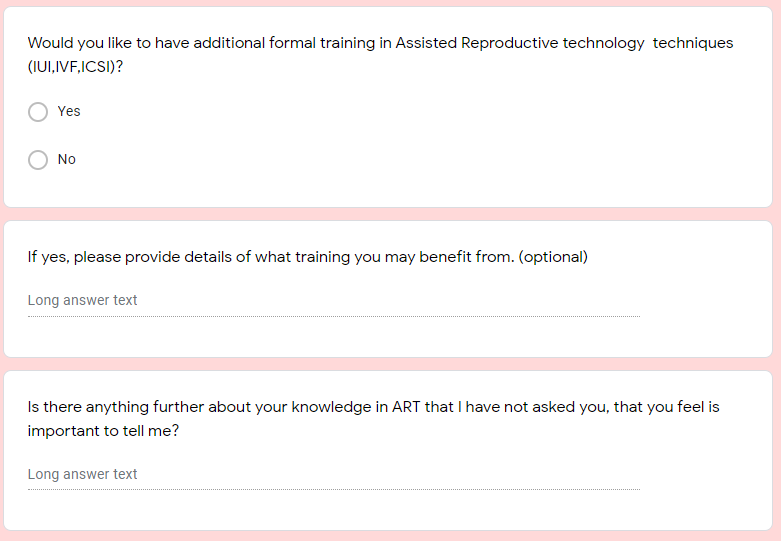


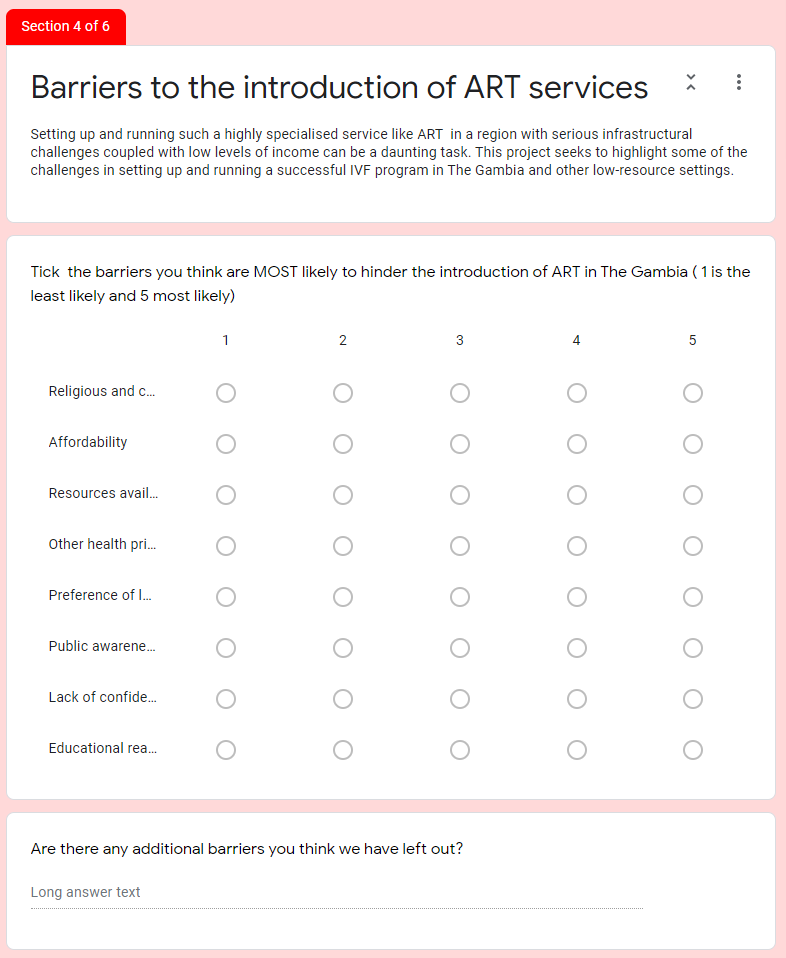


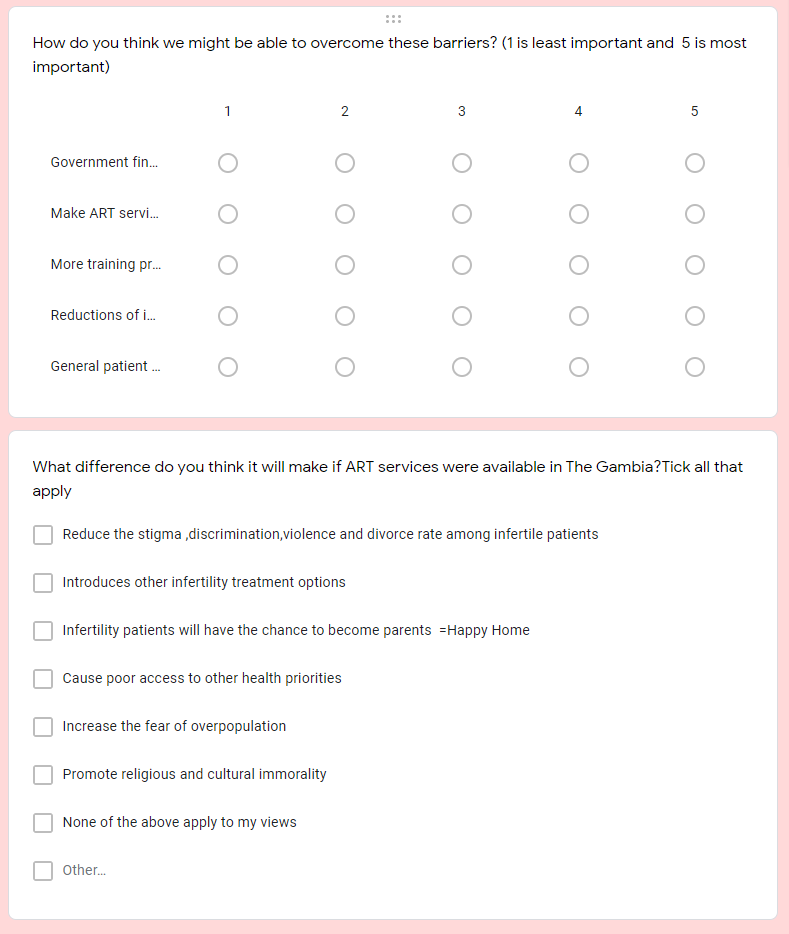


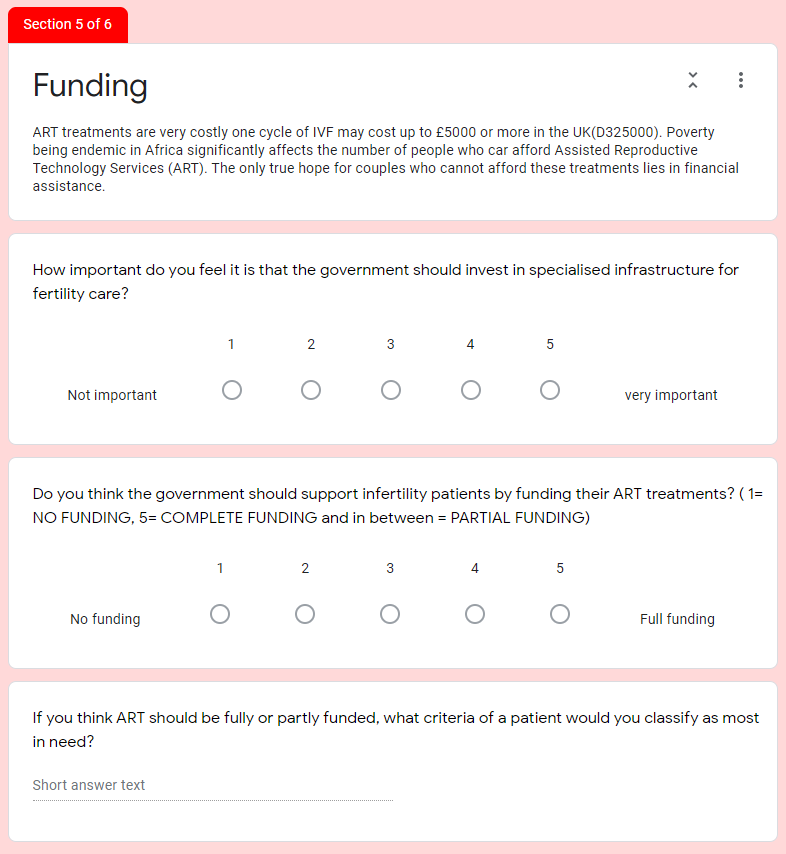


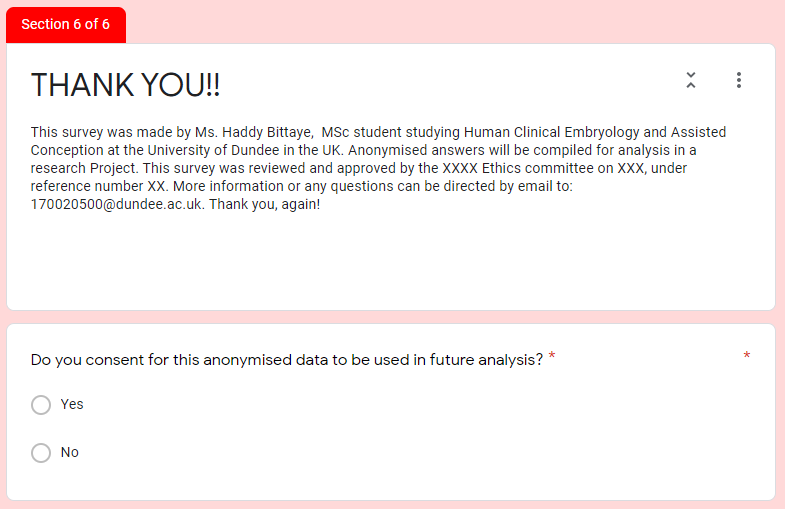


[Forms](https://www.google.com/forms/about/?utm_source=product&utm_medium=forms_logo&utm_campaign=forms)
